# Uncovering Metabolic Alterations in HCT-116 Colon Cancer Cells upon Exposure to Bamboo Leaf Extract Obtained from *Guadua incana* Londoño

**DOI:** 10.3390/molecules29132985

**Published:** 2024-06-23

**Authors:** Luis Carlos Chitiva, Mary Andrea Santamaría-Torres, Paula Rezende-Teixeira, Jessica Rodrigues Pereira de Oliveira Borlot, Rodrigo de Almeida Romagna, Ximena Londoño, Rodrigo Rezende Kitagawa, Leticia V. Costa-Lotufo, Juliet A. Prieto-Rodríguez, Ian Castro-Gamboa, Geison Modesti Costa

**Affiliations:** 1 Grupo de Investigación Fitoquímica Universidad Javeriana (GIFUJ), Department of Chemistry, Faculty of Sciences, Pontificia Universidad Javeriana, Bogotá 110231, Colombia; chitival@javeriana.edu.co (L.C.C.); juliet.prieto@javeriana.edu.co (J.A.P.-R.); 2 Núcleo de Bioensaios, Biossíntese e Ecofisiologia de Produtos Naturais (NuBBE), Institute of Chemistry, São Paulo State University (UNESP), Araraquara 14800-900, Brazil; 3 Universidad de los Andes, Bogotá 111711, Colombia; m.santamariatorres@uniandes.edu.co; 4 Laboratório de Farmacologia de Produtos Naturais Marinhos, Institute of Biomedical Sciences, University of São Paulo, São Paulo 05508-000, Brazil; paularez@usp.br (P.R.-T.); costalotufo@usp.br (L.V.C.-L.); 5 Laboratório de Triagem Biológica de Produtos Naturais, Department of Pharmaceutical Sciences, Federal University of Espírito Santo (UFES), Vitoria 29047-105, Brazil; jessicacarpo@hotmail.com (J.R.P.d.O.B.); rodrigoromagna@gmail.com (R.d.A.R.); rodrigo.kitagawa@ufes.br (R.R.K.); 6 Faculty of Agricultural Sciences, Universidad Nacional de Colombia, Palmira 763533, Colombia; ximelondo@gmail.com

**Keywords:** bamboo leaf extract, *Guadua incana*, cytotoxic activity, HCT-116 cells, endometabolome, exometabolome, anti-inflammatory activity, metabolomics

## Abstract

Metabolic alterations are increasingly recognized as important aspects of colorectal cancer (CRC), offering potential avenues for identifying therapeutic targets. Previous studies have demonstrated the cytotoxic potential of bamboo leaf extract obtained from *Guadua incana* (BLEGI) against HCT-116 colon cancer cells. However, the altered metabolic pathways in these tumor cells remain unknown. Therefore, this study aimed to employ an untargeted metabolomic approach to reveal the metabolic alterations of the endometabolome and exometabolome of HCT-116 cells upon exposure to BLEGI treatment. First, a chemical characterization of the BLEGI was conducted through liquid chromatography coupled with mass spectrometry (LC-MS). Next, we assessed cell viability via MTT and morphological analysis using an immunofluorescence assay against colon cancer cells, and anti-inflammatory activity using an LPS-stimulated macrophage model. Subsequently, we employed LC-MS and proton nuclear magnetic resonance (^1^H-NMR) to investigate intra- and extracellular changes. Chemical characterization primarily revealed the presence of compounds with a flavone glycoside scaffold. Immunofluorescence analysis showed condensed chromatin and subsequent formation of apoptotic bodies, suggesting cell death by apoptosis. The results of the metabolomic analysis showed 98 differential metabolites, involved in glutathione, tricarboxylic acid cycle, and lipoic acid metabolism, among others. Additionally, BLEGI demonstrated significant nitric oxide (NO) inhibitory capacity in macrophage cells. This study enhances our understanding of BLEGI’s possible mechanism of action and provides fresh insights into therapeutic targets for treating this disease.

## 1. Introduction

Cancer is a multiphase process caused by the accumulation of molecular alterations, including the activation of oncogenes, inhibition of tumor suppressor genes, and changes in epigenetic plasticity. These alterations deregulate intracellular signaling pathways, driving the initiation and progression of cancer [1]. The ability of cancer cells to reprogram their metabolism is also critical for survival, growth, and metastasis. Cancer metabolism may involve dysregulated nutrient uptake, intracellular metabolism, gene expression, and interactions with the tumor microenvironment [2,3]. A hallmark and fundamental characteristic of cancer cells lies in their ability to reprogram their metabolism, giving them the ability to undergo uncontrolled proliferation and adapt to various conditions in the tumor environment [4,5]. Unlike normal cells, tumor cells undergo alterations in their cellular metabolism, especially in the way they process glucose and glutamine, as well as an increase in the synthesis of fatty acids. Taking these aspects into account, tumor metabolism has been identified as a therapeutic target in cancer treatment due to the “metabolic adaptability” that cancer cells show in terms of signaling, transporters, and enzymes [6].

Currently, colorectal cancer (CRC) is one of the highest-risk human malignancies and among the most frequently diagnosed cancers. It is a leading cause of death and represents a major global health challenge [7,8,9]. According to projections, deaths from colorectal cancer are expected to increase by 70% worldwide by 2035. Despite advances and the availability of treatments such as radiotherapy, surgery, and chemotherapy, CRC remains the second most common and deadliest malignant disease, with high mortality and morbidity rates [10,11]. This underscores the urgent need to identify therapeutic targets to effectively combat this type of cancer.

In this framework, bamboo plants are still unexplored and promising as valuable sources of bioactive compounds with potential for cytotoxic activity. In recent studies from our research group, we have demonstrated the cytotoxic potential of different species of the genus *Guadua* against colon cancer cells HCT-116. Among them, the bamboo leaf extract obtained from *Guadua incana* presented the best cytotoxic potential with the highest percentage of inhibition (90%) at the maximum concentration evaluated (50 µg/mL) [12]. *Guadua incana* is a woody bamboo native to southeastern Colombia that grows mainly in the humid tropical biome of the eastern slopes of the Andes Mountain range [13]. To date, this species has been subject to few studies on its chemical composition. It has mainly been reported to contain compounds such as glycosylated flavonoids and phenolic acid derivatives [14]. Regarding biological activity, its antioxidant and cytotoxic potential stands out.

Currently, new approaches such as metabolomics have emerged to investigate metabolic alterations, allowing a comprehensive analysis of metabolites present in cells, biofluids, and tissues. Additionally, it is considered a powerful tool to identify metabolic fingerprints associated with exposure to drugs, substances, and plant extracts. Recently, Gao et al., 2016 reported the application of a metabolomics approach to determine metabolic changes in the human colon cell line HCT-116 treated with flexibilide, a diterpene isolated from the soft coral *Sinularia flexibilis*, to explore its probable antitumor mechanism. In this study, 18 significant metabolites involved in different metabolic pathways such as sphingolipid, alanine, aspartate, glutamate, glycerophospholipid, and pyrimidine metabolism were identified. Furthermore, a negative regulation of the tricarboxylic acid cycle and a positive regulation of sphingosine-1-phosphate were found, which could eventually result in cell apoptosis [15].

Another study by Dahabiyeh et al., 2023 used a metabolomic approach to identify the underlying biochemical pathways altered in HCT-116 cells treated with synthesized analogs of 2,3-dihydroquinazolin-4(1*H*)-one. This study revealed that treatment with these compounds induced significant perturbations in different metabolites, including spermine, polyamine, glutamine, creatine, and carnitine, related to biochemical processes essential for cell proliferation and progression, such as biosynthesis and metabolism of amino acids, redox homeostasis, energy-related processes (e.g., fatty acid oxidation, second Warburg-type effect), and one-carbon metabolism [16].

**Figure 1:**
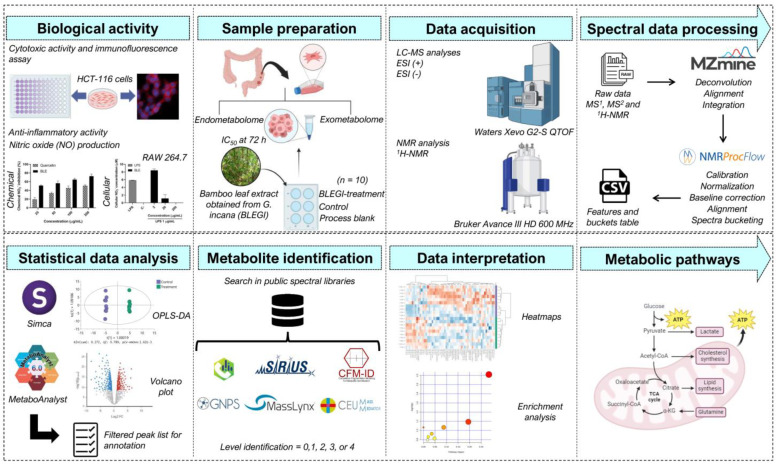
Schematic of the analytical workflow used in this cellular metabolomic study experiment.

To the best of our knowledge, the current study is the first report applying an untargeted metabolomics approach to evaluate metabolic alterations in HCT-116 cells exposed to BLEGI. The study aimed to identify differential metabolites in the endo- and exometabolome of HCT-116 cells after treatment with BLEGI to investigate altered metabolic pathways and gain a first understanding of the possible mechanism of action. The workflow used in this study is summarized in Figure 1.

## 2. Results

### 2.1. Chemical Characterization of Extract

**Figure 2:**
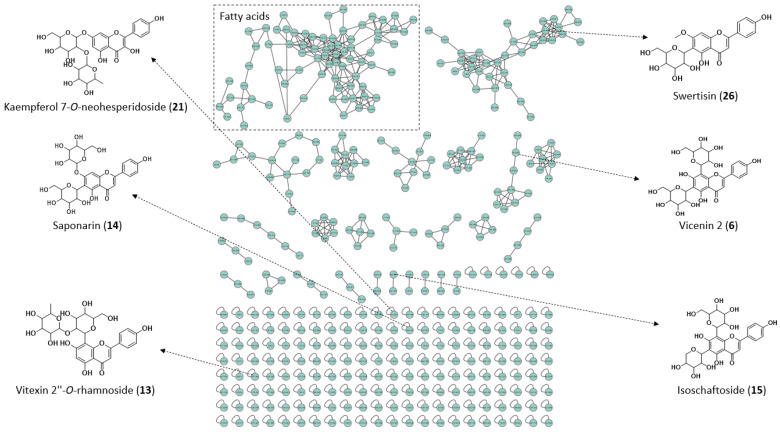
Molecular networking analysis of BLEGI highlighting flavonoids and other compounds, performed using the LC-MS/MS technique in negative mode.

The chemical characterization of BLEGI was carried out by LC-MS/MS in negative and positive ionization modes. The identified metabolites are shown in Table 1 along with their respective levels of identification in accordance with Metabolomics Standards Initiative (MSI) confidence levels [17]. From the BLEGI, a total of 46 compounds were recorded, distributed according to chemical classes, mainly consisting of flavonoids, and cinnamic acid derivatives. Additionally, a mapping of the chemical diversity of BLEGI was carried out using the GNPS online platform, where compounds mainly related to flavonoids were found, including vicenin 2 (**6**), vitexin 2″-*O*-rhamnoside (**13**), saponarin or isosaponarin (**14**), schaftoside or isoschaftoside (**15**), kaempferol 7-*O*-neohesperidoside (**21**), and swertisin (**26**) (Figure 2).

The molecular networking also highlights the presence of other molecular families, corresponding to fatty acids and derivatives for both ionization modes. Particularly in the negative ionization mode, molecular families such as flavonoids and cinnamic acid derivatives are the most abundant for BLEGI. Conversely, in the positive ionization mode, the molecular families of lipids and related molecules were found to a greater extent. The identification of the detected metabolites was based on the search for the main molecular ions (MS) and observed informative fragmentations (MS/MS), along with the use of standards when available, retention data, UV absorption, comparison with literature data, the GNPS web platform, and public databases. The MS/MS spectra for the related metabolites are represented in Figure S1.

From the UV spectra obtained, it was determined that most peaks corresponded to flavonoids, recognized by their characteristic bands and UV absorption maxima. Flavonoids exhibit two absorption maxima due to their two aromatic rings with conjugated double bonds, ring A and ring B. The first band (I) is associated with ring A, with UV absorption occurring between λ_max_ ≈ 300 and 500 nm, while the second band (II) is associated with ring B, which absorbs at between λ_max_ ≈ 240 and 280 nm. Notably, we found that many of the flavonoids corresponded to glycosylated flavone scaffolds, in accordance with reports in the literature, highlighting a range of flavones with absorptions between λ_max_ ≈ 275 and 335 nm [18].

**Table 1:** Metabolites tentatively identified in BLEGI using LC-MS/MS.

No	*t_R_* (min)	UV λ_max_ (nm)	Molecular Formula	Adduct Ion	Calculated Mass (*m*/*z*)	Experimental Mass (*m*/*z*)	Mass Error (ppm)	Main Fragment Ions (MS/MS)	Identification Confidence Level	Metabolite	Reference or Database ^a^
**1**	0.57	-	C_7_H_12_O_6_	[M − H]^−^	191.0551	191.0543	−4.2	175, 147, 129, 111	2	Quinic acid	[14,19,20]
**2**	1.94	Low intensity	C_15_H_16_O_10_	[M − H]^−^	355.0670	355.0669	−0.3	209, 191, 163, 147, 129	4	Not identified	
**3**	2.33	Low intensity	C_16_H_18_O_8_	[M − H]^−^	337.0928	337.0945	5.0	191, 173, 164, 119	2	*O*-Coumaroylquinic acid	[14,18,20,21] and GNPS
**4**	2.36	270, 327	C_27_H_30_O_16_	[M − H]^−^	609.1461	609.1466	0.8	547, 519, 489, 429, 399	3	Luteolin 6-*C*-hexoside 8-*C*-pentoside	
**5**	2.51	274, 336	C_21_H_20_O_10_	[M − H]^−^	431.0983	431.0981	−0.5	387, 341, 311, 179	4	Not identified	
**6**	2.68	272, 325	C_27_H_30_O_15_	[M − H]^−^	593.1511	593.1513	0.3	503, 473, 383, 353, 297	2	Vicenin 2	[14,22] and GNPS
**7**	2.70	269, 325	C_21_H_20_O_11_	[M − H]^−^	447.0932	447.0931	−0.2	327, 299, 285, 133, 109	4	Not identified	
**8**	2.71	268, 337	C_27_H_30_O_15_	[M − H]^−^	593.1511	593.1517	1.0	457, 383, 353, 297	2	Apigenin 6,8-digalactoside	[18] and GNPS
				[M + H]^+^	595.1668	595.1660	−1.3	541, 481, 457, 379, 325, 295			
**9**	2.71	267, 326	C_26_H_28_O_14_	[M − H]^−^	563.1406	563.1411	0.9	473, 443, 383, 353, 325, 2.97	4	Not identified	
**10**	2.74	311	C_9_H_8_O_4_	[M − H]^−^	179.0349	179.0352	1.7	135, 119	1	Caffeic acid ^st^	[23,24,25]
**11**	2.77	271, 333	C_26_H_28_O_14_	[M − H]^−^	563.1406	563.1410	0.7	445, 355, 325, 297	4	Not identified	
**12**	2.80	269, 334	C_26_H_28_O_14_	[M − H]^−^	563.1416	563.1415	−0.2	473, 443, 401, 383, 353, 311	4	Not identified	
**13**	2.87	265, 341	C_27_H_30_O_14_	[M − H]^−^	577.1562	577.1565	0.5	413, 341, 293, 175	2	Vitexin 2″-*O*-rhamnoside	[14] and GNPS
				[M + H]^+^	579.1719	579.171	−1.6	433, 415, 367, 313, 283			
**14**	2.88	269, 342	C_27_H_30_O_15_	[M − H]^−^	593.1511	593.1519	1.3	473, 431, 353, 311, 297, 282	2	Saponarin or Isosaponarin	[14,18] and GNPS
**15**	2.91	271, 335	C_26_H_28_O_14_	[M − H]^−^	563.1406	563.1401	−0.9	473, 443, 383, 353, 325, 297	2	Schaftoside or Isoschaftoside	[14,18,20,21,26,27] and GNPS
				[M + H]^+^	565.1562	565.1563	0.2	547, 529, 499, 457, 427, 379			
**16**	3.13	273, 339	C_26_H_28_O_14_	[M + H]^+^	565.1562	565.1564	0.4	525, 481, 405, 337, 295	3	Vicenin 1 or Vicenin 3	
**17**	3.16	270, 344	C_28_H_32_O_16_	[M − H]^−^	623.1617	623.1617	0.0	504, 443, 353, 323	3	Isoscoparin 2″-*O*-hexoside	
**18**	3.18	271, 342	C_28_H_32_O_15_	[M + H]^+^	609.1824	609.1824	0.0	430, 393, 327, 297, 267	3	Spinosin or Isospinosin	
**19**	3.20	Low intensity	C_9_H_8_O_3_	[M − H]^−^	163.0400	163.0400	0.0	119	1	*p*-Coumaric acid ^st^	[28] and GNPS
**20**	3.23	270, 341	C_27_H_30_O_14_	[M − H]^−^	577.1562	577.1569	1.2	533, 472, 413, 353, 293	2	Violanthin or Isoviolanthin	[29]
**21**	3.24	269, 332	C_27_H_30_O_15_	[M − H]^−^	593.1511	593.1512	0.2	285, 218, 151	2	Kaempferol 7-*O*-neohesperidoside	[29] and GNPS
**22**	3.28	270, 325	C_33_H_40_O_19_	[M − H]^−^	739.2091	739.209	−0.1	593, 431, 281	3	Kaempferol 3-*O*-disaccharoside-7-*O*-pentoside	
**23**	3.28	270, 326	C_27_H_30_O_15_	[M + H]^+^	595.1668	595.1667	−0.2	449, 287	3	Kaempferol 3-*O*-disaccharoside	
**24**	3.35	272, 326	C_21_H_18_O_13_	[M − H]^−^	477.0674	477.0675	0.2	431, 301, 179	3	Quercetin 3-*O*-hexoside	
**25**	3.37	270, 325	C_21_H_20_O_12_	[M − H]^−^	463.0882	463.0880	−0.4	372, 300, 271, 255, 243, 151	2	Hyperoside	[28]
**26**	3.38	271, 328	C_22_H_22_O_10_	[M − H]^−^	445.1140	445.1145	1.1	385, 325, 297, 282, 269	2	Swertisin	[18]
**27**	3.41	271, 329	C_28_H_32_O_17_	[M − H]^−^	639.1566	639.1570	0.6	403, 328, 313, 285, 270, 242	3	Isorhamnetin 3,4′-hexoside	
**28**	3.54	271, 344	C_27_H_30_O_14_	[M − H]^−^	577.1562	577.1561	−0.2	269, 117	3	Apigenin 7-*O*-disaccharoside	
**29**	3.56	269, 342	C_27_H_30_O_14_	[M + H]^+^	579.1719	579.1718	−0.2	433, 363, 271, 153	3	Apigenin 7-*O*-disaccharoside	
**30**	3.58	268, 331	C_22_H_22_O_11_	[M − H]^−^	461.1089	461.1087	−0.4	415, 341, 313, 298	4	Not identified	
**31**	3.59	270, 325	C_28_H_34_O_15_	[M − H]^−^	609.1824	609.1843	3.1	325, 301, 286, 151	3	Hesperidin	
**32**	3.61	271, 344	C_28_H_32_O_15_	[M + H]^+^	609.1826	609.1810	−2.6	463, 301, 286, 258	3	Diosmetin 7-*O*-disaccharoside	
**33**	3.64	269, 326	C_28_H_32_O_16_	[M − H]^−^	623.1617	623.1620	0.5	329, 314, 299, 271, 243, 187	3	Isorhamnetin 3-*O*-hexoside-6″-disaccharoside	
**34**	3.73	265, 341	C_27_H_30_O_15_	[M − H]^−^	593.1511	593.1512	0.2	473, 431, 353, 341, 311, 283	3	Apigenin 6-*C*-hexoside-7-*O*-hexoside	
				[M + H]^+^	595.1668	595.1667	−0.2	415, 379, 337, 313, 283, 165			
**35**	4.47	264, 356	C_15_H_10_O_6_	[M − H]^−^	285.0404	285.0403	−0.4	199, 169, 151, 133	2	Luteolin	[30,31,32]
**36**	4.57	270, 319	C_15_H_12_O_3_	[M − H]^−^	239.0713	239.0699	−5.9	211, 195, 179, 135	3	6-Hidroxyflavanone	
**37**	4.88	264, 329	C_23_H_24_O_12_	[M − H]^−^	491.1195	491.1198	0.6	461, 328, 313, 285, 226	2	Tricin 7-*O*-glucoside	[18]
**38**	4.92	264, 335	C_27_H_32_O_14_	[M − H]^−^	579.1719	579.1720	0.2	271, 177, 151, 119	3	Naringenin-7-*O*-disaccharoside	
**39**	4.95	263, 334	C_21_H_22_O_10_	[M − H]^−^	433.1140	433.1145	1.2	271, 151, 119	3	Naringenin-7-*O*-hexoside	
**40**	4.97	271, 358	C_15_H_10_O_5_	[M − H]^−^	269.0455	269.0460	1.9	227, 181, 151, 117	2	Apigenin	[30]
**41**	5.01	244, 315	C_15_H_10_O_6_	[M − H]^−^	285.0404	285.0405	0.4	211, 183, 149, 121	3	3,6,2′,3′-Tetrahydroxyflavone	
**42**	5.03	245, 319	C_15_H_10_O_5_	[M − H]^−^	269.0455	269.0461	2.2	239, 211, 187, 143, 117	3	6,7,4′-Trihydroxyisoflavone	
**43**	5.04	269, 354	C_15_H_10_O_6_	[M − H]^−^	285.0404	285.0420	5.6	255, 227, 187, 159, 143, 117	2	Kaempferol	[29,30]
**44**	5.09	245, 319	C_17_H_14_O_7_	[M − H]^−^	329.0666	329.0671	1.5	271, 243, 227, 161, 133	3	4′,5,7-Trihydroxy-3,6-dimethoxyflavone	
**45**	5.12	244, 325	C_16_H_12_O_6_	[M − H]^−^	299.0561	299.0561	0.0	256, 227, 212, 183, 151	3	3,5,7-Trihydroxy-4′-methoxyflavone	
**46**	5.15	244, 354	C_17_H_14_O_7_	[M − H]^−^	329.0666	329.0669	0.9	229, 271, 227, 215, 161	2	Tricin	[30]
				[M + H]^+^	331.0823	331.0820	−0.9	313, 285, 270, 258, 203, 153			

^a^ The references described compounds found in the literature for leaves, culms, and shoots of several bamboo plants and database sources such as PubChem, METLIN, KEGG, and GNPS; ^st^ The identification of metabolites has been verified by using authentic standards; *m*/*z* data were obtained by LC-MS based on time and peak areas. λ_max_: UV maximum absorbance wavelength. Metabolites were annotated with identification confidence levels as recommended by the Metabolomics Standards Initiative (MSI) according to the following: Level 0: Unambiguous 3D Structure: isolated, pure compound, including full stereochemistry; Level 1: Confident 2D structure: uses reference standard match or full 2D structure elucidation; Level 2: Probable structure: matched to literature data or databases by diagnostic evidence, Level 3: Possible structure or class: most likely structure, isomers possible, substance class or substructure match; Level 4: Unknown feature of interest [17].

### 2.2. Cell Viability

**Figure 3:**
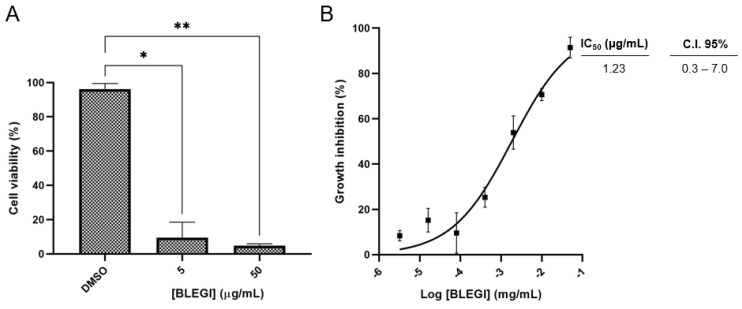
(**A**) Cell viability after 72 h incubation at different concentrations. (**B**) Concentration–response curves of the cytotoxicity of HCT-116 cells after 72 h treatment with the BLEGI using concentrations of 0.0032 to 50 μg/mL. The data represent the mean of three independent biological replicates with at least technical duplicates ± SEM (standard error of the mean). All experiments employed 0.05% DMSO (vehicle) and doxorubicin (DOXO) ranging from 0.00064 to 10 µM with IC_50_ = 0.2 µM (C.I 95% 384 0.14–0.25) as negative and positive controls, respectively. One-way ANOVA analysis using Tukey’s post hoc test was employed to indicate significance, denoted as follows: * *p* < 0.05 and ** *p* < 0.01 compared to DMSO.

We evaluated the cytotoxic effect of BLEGI at two concentrations (5 and 50 µg/mL) to determine the percentage of cell viability against the HCT-116 colon cancer cell line. We found that at a concentration of 5 μg/mL, it presented a cell viability percentage of 10%, while at 50 µg/mL, it presented a cell viability percentage of 5% (Figure 3A). Considering these results, the IC_50_ value was determined in a concentration range of 0.0032 to 50 µg/mL. BLEGI revealed significant cytotoxicity against the colon cell line with an IC_50_ value of 1.23 µg/mL. Figure 3B shows the concentration–response curve of cytotoxicity of HCT-116 cells after 72 h of BLEGI treatment. Therefore, the IC_50_ value was used for the treatment of cells in the metabolomics study.

### 2.3. Immunofluorescence Assay

**Figure 4:**
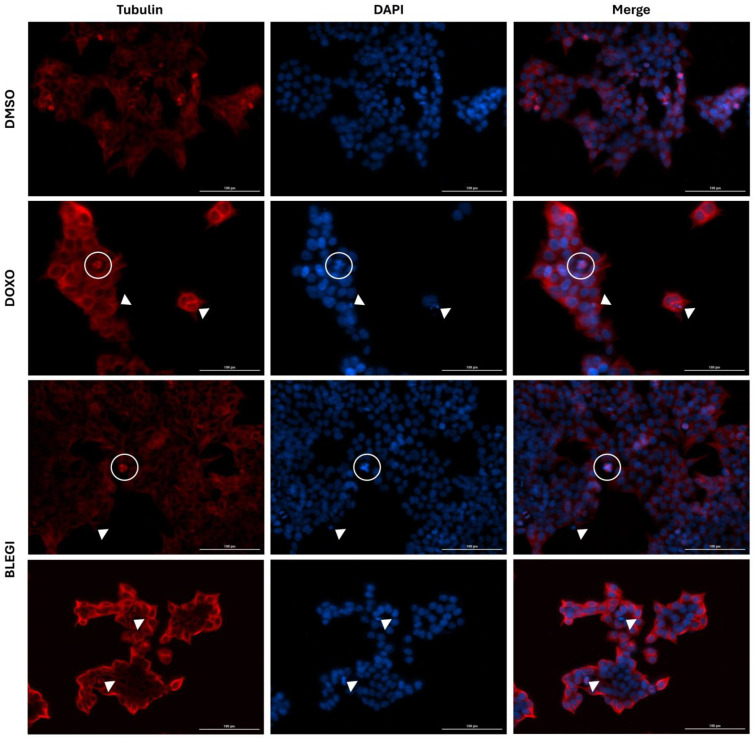
Immunofluorescence of α-tubulin and DAPI in HCT 116 cells after 72 h of incubation with DMSO 0.1%, DOXO 0.2 µM, and BLEGI 1.23 µg/mL. Arrows indicate condensed chromatin (suggestive of apoptosis) and circles indicate tripolar mitosis.

To determine whether BLEGI induced apoptosis in colon cancer cells, the α-tubulin antibody was evaluated, and cellular staining with DAPI was performed to examine cell morphology by immunofluorescence analysis. Treatment of HCT-116 cells with BLEGI significantly induced the formation of apoptotic bodies, and a tripolar mitosis process was also observed, unlike the control used, DMSO. After 72 h of treatment, the cytoskeleton of the cells (red) was observed with α-tubulin, while the cell nuclei (blue) and apoptotic bodies were observed with DAPI. By merging the images, the cellular processes involved are clearly observed (Figure 4). Doxorubicin was used as a positive control. These results imply that BLEGI can induce apoptosis of colon cancer cells.

### 2.4. Nitric Oxide (NO) Production Inhibition

**Figure 5:**
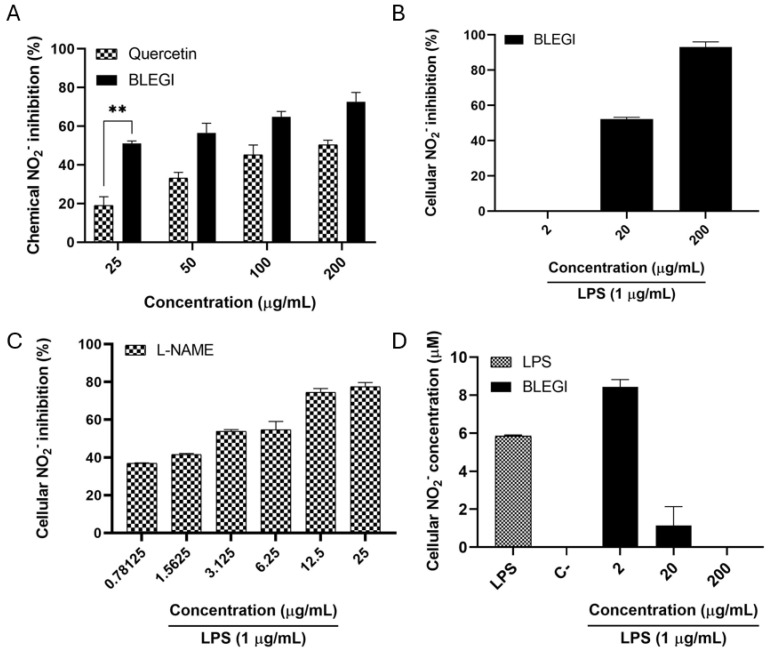
(**A**) Percentage of chemical NO_2_^−^ after treatment with BLEGI, evaluated at four concentrations (25, 50, 100, and 200 µg/mL). (**B**) Percentage of cellular NO_2_^−^ in the murine cell line RAW 264.7 after treatment with BLEGI, evaluated at three concentrations (2, 20, and 200 µg/mL). (**C**) Level of cellular NO_2_^−^ in the murine cell line RAW 264.7 after treatment with L-NAME used as an inhibition control. (**D**) Cellular NO_2_^−^ levels in the murine RAW 264.7 cell line stimulated with LPS after treatment with BLEGI, evaluated at three concentrations (2, 20, and 200 µg/mL). One-way ANOVA analysis using Tukey’s post hoc test was employed to indicate significance, denoted as follows: ** *p* < 0.01 compared to Quercetin.

To determine the percentage inhibition of NO by BLEGI, two methods were employed: first, the percentage inhibition of chemical NO was determined using the spectrophotometric method; and second, the percentage inhibition of cellular NO was determined in LPS-stimulated macrophages. Figure 5A shows the effect of BLEGI on the inhibition of NO. It is observed that at the four concentrations evaluated (25, 50, 100, and 200 µg/mL), it presented inhibition percentages greater than 50%, indicating a significant potential. Cell viability was first examined by 3-(4,5-dimethylthiazol-2-yl)-2,5-diphenyltetrazolium bromide (MTT) assay to exclude false positive results caused by possible cytotoxicity of BLEGI. The cytotoxicity of BLEGI was also evaluated in the macrophage cell line, where no cytotoxic effect was observed at the concentrations evaluated (50 to 200 µg/mL) since the IC_50_ value of BLEGI was greater than 200 µg/mL in all concentrations analyzed. Figure 5B shows the effect of BLEGI on the production of NO in LPS-stimulated macrophages. It should be noted that, at the maximum concentration evaluated, it exhibits an inhibition percentage greater than 90%, indicating powerful inhibition of NO compared to the positive control. For the NO production assessed in macrophage inhibition tests, concentrations of 200 µg/mL, 20 µg/mL, and 2 µg/mL were used. Moreover, N(ω)-nitro-l-arginine methyl ester (L-NAME), an inhibitor of NO synthase, was used as a positive control (Figure 5C). Figure 5D displays the results of cellular NO levels in LPS-stimulated macrophages after BLEGI treatment. BLEGI showed significant inhibitory effects against NO production, with values of 8.44 ± 0.38 μM at a concentration of 2 µg/mL, comparable to the positive control.

### 2.5. Untargeted Endo- and Exometabolome Analysis

To explore the metabolic profile of BLEGI-treated HCT-116 cells, we employed an untargeted metabolomics approach using LC-MS and ^1^H-NMR analyses. To elucidate the metabolic differences between BLEGI-treated and untreated cells, the endo- and exometabolome of the cells were profiled using the two analytical platforms. Multivariate data analysis was conducted on the preprocessed data utilizing unsupervised principal component analysis (PCA) and supervised orthogonal partial least squares discriminant analysis (OPLS-DA) models. The quality and stability of the endo- and exometabolome profiling instrument were assessed by performing PCA of the quality control (QC) and samples. Figure S2 illustrates the groupings of the QC samples for each of the analytical platforms used, indicating the reliability and validity of the data, respectively. Also, we observed notable separation trends between BLEGI-treated and untreated cells on the two platforms, indicating significant differences in the metabolites of HCT-116 cells after treatment. Additionally, all experimental clustering samples in the PCA score plots were within the 95% confidence interval.

**Figure 6:**
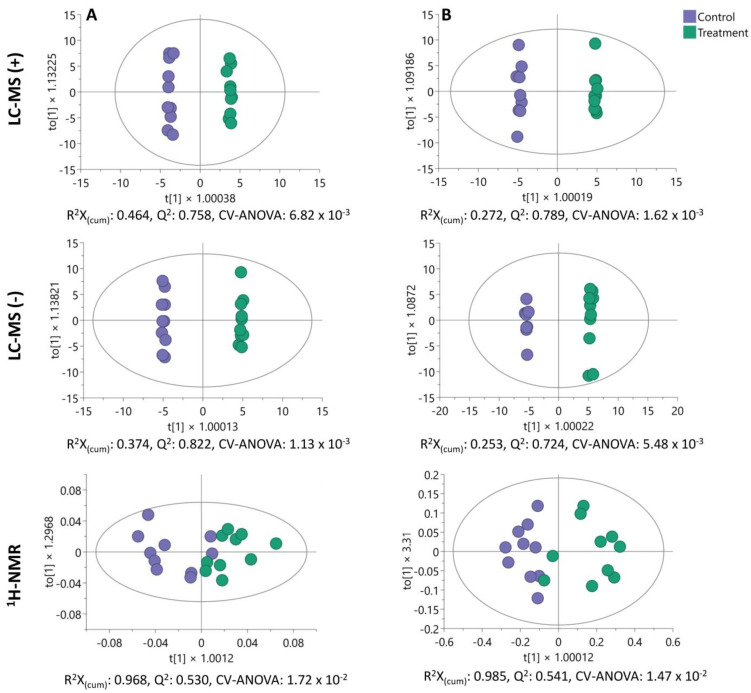
OPLS-DA score plots comparing the BLEGI-treated cells (green) with the untreated ones (purple) for LC-MS and ^1^H-NMR data of the (**A**) endometabolome and (**B**) exometabolome of HCT-116 cells.

Furthermore, we built OPLS-DA models between the groups of BLEGI-treated and untreated cells, showing good quality parameters and complete separation of the groups for the endometabolome (Figure 6A) and exometabolome (Figure 6B), respectively. This allowed us to identify the significant variables contributing to the separation between the groups. Subsequently, to validate the analysis, we used the permutation test to assess the reliability of the OPLS-DA model by analyzing the values of R^2^ and Q^2^. Figure S3 shows the permutation test plots (*n* = 200) of the OPLS-DA models for BLEGI-treated and untreated cells. For all OPLS-DA models, the values of R^2^ and Q^2^ demonstrated good robustness without overfitting, indicating that the OPLS-DA model can effectively distinguish each group of samples.

### 2.6. Analysis of Altered Metabolites in HCT-116 Cells

**Figure 7:**
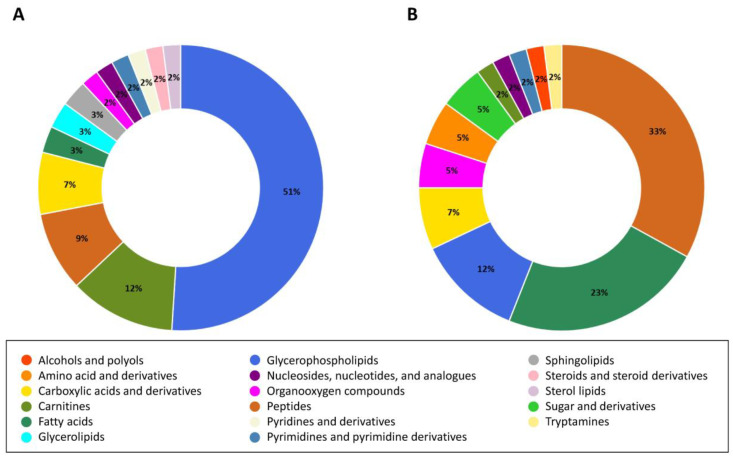
Altered (**A**) endo- and (**B**) exometabolome metabolites after treatment with BLEGI in HCT-116 cells. Chemical classes are shown according to percentage and color.

A volcano plot was generated to identify the significantly differential metabolites in the endo- and exometabolome of HCT-116 cells. The differential metabolites were selected based on criteria such as variable of importance in the projection (VIP) > 1, fold change (FC) > 1.2, or FC < 0.5, and *p*-value corrected with false discovery rate (FDR) < 0.05 (Figure S4). The metabolites were identified according to the Metabolomics Standards Initiative [17]. A total of 57 altered metabolites were detected in the endometabolome of BLEGI-treated cells, with 14 upregulated and 43 downregulated. Figure 7A shows the differential metabolites of the endometabolome corresponding to different classes including glycerophospholipids (51%), carnitines (12%), peptides (9%), carboxylic acids and derivatives (7%), fatty acids (3%), glycerolipids (3%), sphingolipids (3%), organooxygen compounds (2%), nucleosides, nucleotides, and analogs (2%), pyrimidines and pyrimidine derivatives (2%), pyridines and derivatives (2%), steroids and steroid derivatives (2%), and sterol lipids (2%). A total of 41 altered metabolites were detected in the exometabolome of BLEGI-treated cells, with 26 upregulated and 15 downregulated. Figure 7B shows the differential metabolites identified in the exometabolome corresponding to different classes, including peptides (33%), fatty acids (23%), glycerophospholipids (12%), carboxylic acids and derivatives (7%), organooxygen compounds (5%), amino acid and derivatives (5%), sugar and derivatives (5%), carnitines (2%), nucleosides, nucleotides, and analogs (2%), pyrimidines and pyrimidine derivatives (2%), alcohols and polyols (2%), and tryptamines (2%). Tables S1 and S2 show detailed information about the metabolites found.

To confirm the metabolic alterations caused by BLEGI treatment in HCT-116 cells, ^1^H-NMR-based metabolomic analyses were performed. All spectra were analyzed for integration and identification of metabolites that showed a significant change using MestReNova v14.2.0 software (Mestrelab Research S.L., Santiago de Compostela, Spain) and comparison with different reports in the literature and open databases. Figure S5A,B show representative ^1^H-NMR spectra for the endo- and exometabolome of BLEGI-treated HCT-116 cells. The spectrum of the exometabolome (Figure S5A) shows significant changes in the region with a chemical shift for protons (δ_H_) at δ_H_ 3.65 and 4.85 ppm, specifically for the signals corresponding to the metabolites glutathione (GSH) and glutathione disulfide (GSSH), which showed the greatest changes, with an increase and decrease, respectively. In the region of δ_H_ from 2.00 to 3.00 ppm (which is considered most relevant for GSH and GSSH), we were able to identify these metabolites based on the signals at 2.91, 2.10, 2.36, and 2.97 ppm, respectively. Additionally, with the ^1^H-NMR analysis, it was possible to extract specific signals corresponding to derivatives of organic acids and fatty acids for the endo- and exometabolome, respectively. The signals for GSH stand out with δ_H_ 2.91 (dd), 2.36 (m) and δ_H_ 4.46 (q), 3.86 (m), respectively, confirming the identification of this metabolite. In the exometabolome spectrum (Figure S5B), the changes were not so obvious. However, in the region with δ_H_ 0.95 to 1.55 ppm, some specific signals for fatty acids are shown, with these metabolites presenting significant decreases. However, it is important to highlight that the ^1^H-NMR spectra obtained present a considerable overlap of signals, considering that it is a complex extract. Despite this, several signals were identified that confirmed the presence of the metabolites detected by LC-MS (Tables S1 and S2).

**Figure 8:**
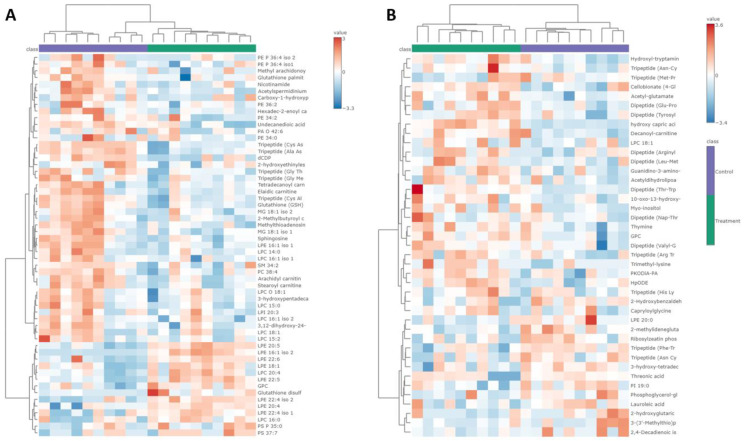
Hierarchical clustering with a heatmap illustrating the differences in the abundance of metabolites found in (**A**) endometabolome and (**B**) exometabolome between BLEGI-treated cells and untreated cells. The x-axis shows the clustering of all samples, and the y-axis shows the clustering of the metabolites found for the endo- and exometabolome of HCT-116 cells.

The hierarchical clustering analysis and the heatmap based on the annotated metabolites comparing BLEGI-treated and untreated cells showed significant changes, generating a visualization of the distribution of the metabolites found. We highlight that the metabolites that presented a significant change with an FC greater than 1.2 in the group of BLEGI-treated cells were lysophosphatidylethanolamines (LPE) and phosphatidylserines (PS), while lysophosphatidylcholines (LPC), phosphatidylinositol (PI), and phosphatidylethanolamines (PE) presented a significant change with an FC less than 0.5. It was determined that each group shares different patterns in terms of metabolic composition and levels of presence and abundance. A heatmap showing the top 40 metabolites altered in BLEGI-treated cells is presented in Figure 8A,B. Among the significantly decreased metabolites were fatty acyls, carboxylic acid derivatives, PE, PI, and amino acid derivatives.

**Figure 9:**
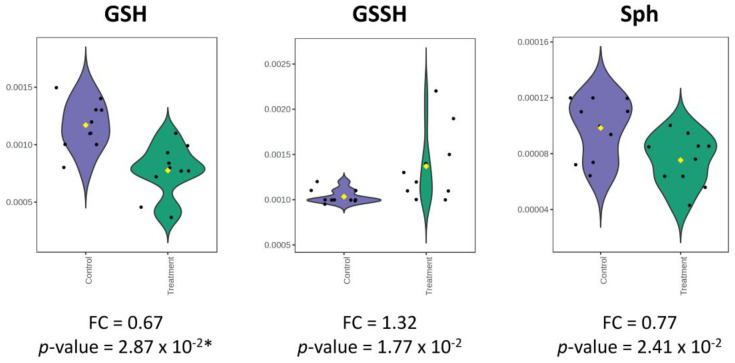
Violin plot for the altered metabolites corresponding to BLEGI-treated and untreated cells. Y-axes are represented as relative units. The date was normalized with respect to the total spectral area. Boxes range from the 5% and 95% percentiles are indicated as error bars; individual data points are indicated by circles. The medians are indicated by horizontal lines within each box. * Metabolites corrected with FDR value.

Violin plots show the statistically significant and differential metabolites for BLEGI-treated and untreated cells. In Figure 9, we present the changes in the main metabolites that exhibited a significant change. We found that for the endometabolome, glutathione (GSH) (FC = 0.67; FDR-adjusted *p*-value = 2.87 × 10^−2^), glutathione disulfide (GSSH) (FC = 1.32; *p*-value = 1.77 × 10^−2^), and sphingosine (Sph) (FC = 0.77; *p*-value = 2.41 × 10^−2^) exhibited significant changes. Our study also highlighted the importance of other metabolites with significant changes, which are described in Tables S1 and S2.

### 2.7. Exploration of Altered Metabolic Pathways in HCT-116 Cells

**Figure 10:**
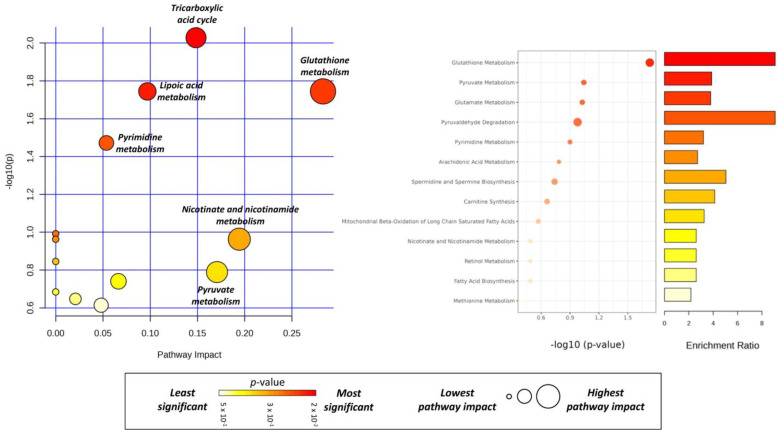
Metabolic pathway analysis based on the enrichment analysis procedure for metabolites found in the metabolome of HCT-116 cells after exposure to BLEGI. The most relevant biosynthesis pathways are represented according to *p*-value < 0.05 and pathway impact (pi) > 0. (1) Glutathione metabolism (*p*-value = 0.022, pi = 0.282), (2) tricarboxylic acid cycle (*p*-value = 0.010, pi = 0.148), (3) lipoic acid metabolism (*p*-value = 0.021, pi = 0.096), and (4) pyrimidine metabolism (*p*-value = 0.039, pi = 0.053). Summary pathways biosynthesis identified in endo- and exometabolome generated with MetaboAnalyst 6.0 based on differential metabolites.

Based on the observed variations in the metabolites found above, and to further elucidate the effect of BLEGI treatment on the metabolic processes of HCT-116 cells, it was necessary to explore the profoundly altered metabolic pathways. In this regard, the annotation of the differential pathways of the metabolites was performed using the KEGG database. MetaboAnalyst was employed to identify the altered metabolic pathways and elucidate the metabolic differences between the BLEGI-treated and untreated cells. Different target metabolic pathways were found to be altered in HCT-116 cells. Through this analysis and as shown in Figure 10, possible pathways altered with BLEGI treatment included glutathione metabolism (*p*-value = 0.022, pi = 0.282), tricarboxylic acid cycle (*p*-value = 0.010, pi = 0.148), lipoic acid metabolism (*p*-value = 0.021, pi = 0.096), and pyrimidine metabolism (*p*-value = 0.039, pi = 0.053), among others. These metabolic pathways were found to be the most affected and the most influential in the enrichment analysis of HCT-116 cells after BLEGI exposure.

## 3. Discussion

In the present study, the metabolome of HCT-116 cells was analyzed after the effect induced by BLEGI, using a metabolomic approach by LC-MS and ^1^H-NMR untargeted of the endometabolome and exometabolome. Primarily, the cytotoxic effect of BLEGI on HCT-116 cells was determined considering an exposure time of 72 h. The results showed a potent cytotoxic effect of BLEGI on HCT-116 cells with an IC_50_ value of 1.23 µg/mL. These results were comparable to the ethanol extract obtained from the species *G. incana* in a previous study carried out on the same cells [12]. In this regard, for the metabolomic analysis, the HCT-116 cells were treated with the IC_50_ value obtained from bamboo leaf extract.

**Figure 11:**
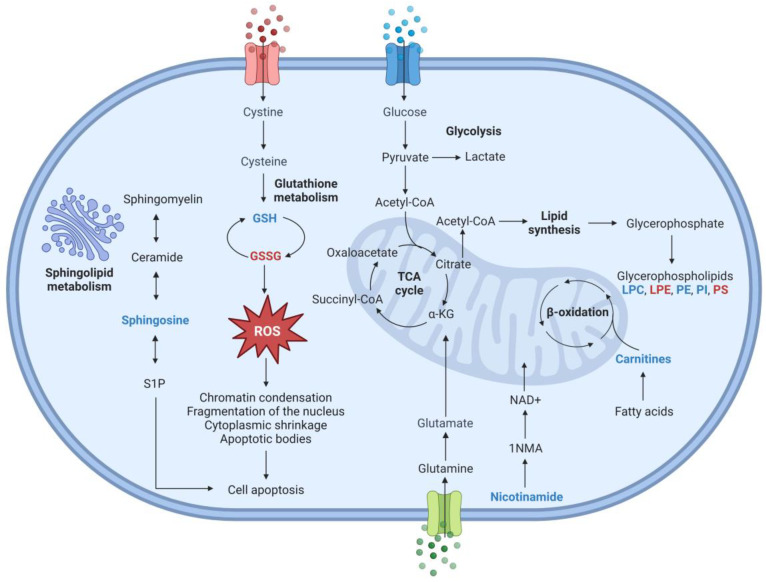
Schematic biochemical representation of the main altered metabolic pathways 72 h after BLEGI treatment in HCT-116 cells. Red indicates potential upregulated metabolites, while blue indicates potential downregulated metabolites.

Metabolomic analysis of the endo- and exometabolome of HCT-116 colon cancer cells demonstrated significant changes in several metabolites involved in various affected metabolic pathways. Among the main metabolic pathways affected, glutathione metabolism, tricarboxylic acid cycle, lipoic acid metabolism, and pyrimidine metabolism stand out. When comparing the BLEGI-treated group with the untreated group, several metabolites associated with glutathione metabolism exhibited a notable impact value of 0.282. Metabolites produced in the context of glutathione metabolism comprised both reduced GSH and GSSG. The glutathione metabolism pathway plays a crucial role in intracellular antioxidant defense, redox regulation, and detoxification. This is achieved through the dynamic interaction of the GSH–GSSG redox pair together with various enzymes and glutathione-dependent transporter systems. GSH, which contains a crucial functional thiol group, functions as a key intercellular antioxidant by neutralizing free radicals and reactive oxygen compounds. It also acts by reducing oxidized or inactivated protein thiols to preserve their biological activities. Consequently, GSH is considered a vital indicator of oxidative stress [33,34]. As shown in Figure 11, GSH showed a decrease in BLEGI-treated cells compared to the untreated cells, while GSSG showed an increase. These findings suggest that BLEGI influenced cellular redox status. The marked reduction in glutathione levels implies a potentially diminished ability to maintain intercellular redox homeostasis. Studies have shown a close relationship between GSSG levels and loss of mitochondrial integrity and caspase activation [35]. The adverse effects of BLEGI-induced oxidative stress could play a critical role in inhibiting cell proliferation, thus demonstrating its antitumor activity. Reactive oxygen species (ROS) are generated as byproducts of regular redox reactions that occur during cellular metabolism. However, when ROS levels exceed normal thresholds and are not efficiently removed, oxidative stress occurs, resulting in significant metabolic alterations and cellular damage. Elevated levels of ROS can interact with essential biomolecules such as lipids, nucleic acids, and proteins, disrupting their normal functions and contributing to chronic diseases, particularly colorectal cancer [36,37]. Under physiological conditions, there is a delicate balance between the antioxidant and pro-oxidant systems. Protective mechanisms include the glutathione system, where glutathione exists primarily in its reduced form GSH within cells, with a smaller fraction in its oxidized state GSSG. This balance is maintained by the enzyme glutathione reductase (GR), which converts GSSG back to GSH, ensuring cellular redox homeostasis [38,39]. Surprisingly, very low levels of GSH in cells treated with BLEGI compared to other studies demonstrate that high levels of GSH in tumor cells are associated with cancer progression and increased resistance to chemotherapeutic drugs. Several novel therapies targeting the GSH antioxidant system have recently been developed in different tumors as a means to increase response and decrease drug resistance [40]. The decrease in GSH in HCT-116 cells treated with BLEGI strongly supports the hypothesis of glutathione metabolism as a possible pathway responsible for the activity of the extract. This is coherent with what was observed in human liver HepG2 cells treated with a flavonoid derivative, where the low level of GSH in the treated cells indicated an increase in oxidative stress [34].

Nicotinamide (NAM) serves as a precursor to nicotinamide-adenine dinucleotide (NAD^+^), which plays crucial roles in both redox and non-redox reactions regulating cellular energy metabolism. NAD^+^ functions as a coenzyme in dehydrogenase reactions, facilitating the production of adenosine triphosphate (ATP). Elevated levels of NAD^+^ inhibit the generation of ROS and enhance mitochondrial function, thereby mitigating oxidative stress and promoting cell viability. Moreover, NAD^+^ serves as a pivotal metabolite in cellular homeostasis, acting as an essential cofactor in redox reactions involved in various energy production pathways, including fatty acid oxidation, the Krebs cycle, and glycolysis [1,41,42]. Nicotinamide N-methyltransferase (NNMT) is an enzyme found in the cytoplasm that catalyzes the methylation of NAM and related compounds like pyridines [43,44]. In the background of HCT-116 tumor cell metabolism, this information could be relevant, since NNMT and its metabolic products could play a role in the regulation of certain metabolic aspects or specific pathways in these cells. NNMT and its metabolic products have been studied in the context of various diseases, including some types of cancer, although the precise connection with the tumor-specific metabolism of HCT-116 may require further investigation.

Dysregulated lipid metabolism is a significant metabolic alteration in cancer. Lipids encompass a complex group of biomolecules, including fatty acids, glycerides, non-glyceric lipids, and lipoproteins. Their diverse roles include serving as energy producers, signaling molecules, and source material for cell membrane biogenesis. Lipid metabolism generates various biological intermediates, many of which act as signaling molecules, regulating pathways controlling growth, proliferation, differentiation, apoptosis, motility, inflammation, survival, and cell membrane homeostasis [45,46]. A comprehensive understanding of how cancer cells reprogram lipid metabolism to support their malignant phenotype may unveil new therapeutic targets for cancer treatment. In our study, sphingosine (Sph) levels decreased after BLEGI treatment, possibly due to cell membrane damage in HCT-116 cells. The cells then relied on sphingolipids to enhance membrane synthesis. The results suggest that BLEGI may regulate apoptosis and the dysregulated metabolism of sphingolipids to exert its antitumor effect. Sphingolipids are essential molecules that comprise cell membranes and act as second messengers participating in numerous cellular functions, such as cell signaling, proliferation inhibition, cell cycle arrest, induction of apoptosis (programmed cell death), stress responses, necrosis, inflammation, senescence, and differentiation in various types of cancer cells [47,48,49]. In this study, the results revealed an intracellular decrease in Sph, which could lead to the inhibition of cell growth and induction of apoptotic cell death in HCT-116 cells after BLEGI treatment. Sph has been implicated in various signaling pathways involved in the apoptotic process and acts as a second messenger in response to various cellular stress signals, such as DNA damage or cellular injury. Elevated levels of Sph promote cell cycle arrest and apoptosis, preventing uncontrolled cell growth [50]. Therefore, these findings suggest that BLEGI-induced apoptosis of HCT-116 colon cancer cells could be due to the reduction in Sph levels in the endometabolome. Previous studies have suggested that Sph plays a specific role in ceramide-independent tumor cell death [51].

We present a comprehensive biochemical map illustrating the metabolites and metabolic pathways altered by BLEGI in HCT-116 colon cancer cells (Figure 11). Our findings reveal that BLEGI significantly impacts major metabolic pathways, including glutathione metabolism, the tricarboxylic acid cycle, lipoic acid metabolism, and pyrimidine metabolism. The endo- and exometabolomic profile of HCT-116 cells exhibits marked changes in metabolite concentrations following a 72 h exposure to BLEGI. The integrated analysis allows us to suggest that BLEGI treatment may induce cell death by apoptosis. To confirm the findings regarding the induction of apoptosis in HCT-116 cells and its impact on the mechanism of action, we evaluated cell morphology and qualitative apoptosis induction using an immunofluorescence assay. Through this analysis, we obtained qualitative information about cell structure and processes. In this assay, we used the antibody α-tubulin, which is one of the main cytosolic fibers constituting the cytoskeleton. Additionally, we used DAPI staining to visualize nuclear DNA in the cells and evaluate their macroscopic cell morphology. Notably, our immunofluorescence analysis revealed the presence of tripolar mitosis, a process of cell division driven by molecular mechanisms typical of mitosis. Additionally, we observed the formation of apoptotic bodies, which are eventually phagocytosed, indicating an effect directly related to apoptotic cell death [52] in HCT-116 cells after BLEGI treatment. These results highlight the promising therapeutic potential of BLEGI not only for colon cancer but also for other types of cancer. Considering that flavonoids, specifically flavone glycosides, are responsible for the cytotoxic effect on HCT-116 cells, in a previous study, a bioinformatic analysis and complete transcriptomic profile were performed with the aim of revealing regulatory pathways associated with cell apoptosis induced by quercetin in HCT-116 cells. It was determined that cell viability was markedly reduced with this flavonoid as a function of dose and time, respectively. Furthermore, it was confirmed that quercetin could inhibit proliferation and induce apoptosis. This could be associated with the transcriptome of HCT-116 cells, considering the differential expression of ncRNA and mRNA. Twelve deregulated ncRNAs and mRNAs were found related to two significantly enriched pathways, the PI3K-Akt and Ras signaling pathways, confirming that they may be related to the molecular mechanisms that quercetin exerts on CRC [53]. These findings could help us elucidate the molecular mechanism role of BLEGI flavone glycosides and possible alterations in HCT-116 cells; however, further studies are required.

Furthermore, the anti-inflammatory activity of BLEGI was determined by calculating the percentage of inhibition of NO at the chemical and cellular levels in macrophage cells. Generally, BLEGI showed significant results for the inhibition of NO production, with an inhibition greater than 50%, which is considered an important result considering that NO is a factor involved in tumor progression. Considering the results of chemical NO and cellular NO, it is possible to infer that the samples can capture NO and inhibit iNOS (inducible nitric oxide synthase). The relating of metabolomic analysis results to anti-inflammatory activity allows us to highlight a close relationship between inflammatory processes and tumor development. Thus, inflammation plays an important role in the development of different types of cancer. The enzyme iNOS is one of the three enzyme isoforms of nitric oxide synthase (NOS) being an enzyme expressed under inflammatory conditions that can produce high levels of NO, affecting the redox state of cells causing protein oxidation, lipid peroxidation, and DNA damage. Thus, several iNOS inhibitors have shown efficacy in reducing metastasis and inhibiting tumor cells [54,55,56]. Our hypothesis is that BLEGI may present a relevant anti-inflammatory capacity targeting iNOS and the regulation of oxidative stress, being a potential preventive agent against the growth of these tumor cells.

Based on the results of NO inhibitory activity of BLEGI, it has been reported that flavonoids normally have NO inhibitory activity [57]. The chemical characterization of this extract allowed us to determine that phenolic compounds are the major compounds in BLEGI. The presence of glycosylated flavonoids and phenolic acids and derivatives stands out [14]. Consequently, and according to the reports described above, it is established that flavonoids present anti-inflammatory and immunomodulatory properties in different tissues. Since NO produced by iNOS is one of the inflammatory mediators in cells, flavonoids can inhibit NO production, measured at the chemical and cellular levels. NO production is a primary indicator of macrophage activation. NO is produced once the nuclear factor kappa B (NF-κB) complex has been activated by extracellular stimuli [58]. Therefore, BLEGI, which suppresses NO production in macrophage cells, can be expected to be a useful suppressor of inflammation [58]. Flavonoids can inhibit the synthesis and activities of different proinflammatory mediators (e.g., cytokines, chemokines, NO, eicosanoids, etc.), and also have the ability to inhibit transcription factors such as nuclear factor kappa B (NF-κB) [59,60]. Our study constitutes the first report on the anti-inflammatory properties of BLEGI. The results described in this study validate that the *G. incana* species is a remarkable source of bioactive compounds with important biological properties related to its anti-inflammatory and antioxidant capabilities.

Nevertheless, some limitations of our study should be noted. First, specific biological assays are needed to validate the mechanism of action in HCT-116 cells after BLEGI exposure. It is additionally recommended to continue performing tests specifically aimed at confirming the mechanism of cell death (e.g., assays focused on glutathione determination, quantitative apoptosis assay (Annexin V), glucose uptake, mitochondrial membrane potential, and evaluation of ROS levels). It is also worth noting that more research is needed to validate other potential therapeutic targets and fully understand the mechanism by which BLEGI exerts its effect. In summary, we identified multiple altered biological pathways after exposure to BLEGI, which shows us the possible altered metabolic pathways and provides insight into the possible mechanism of action in these tumor cells.

## 4. Materials and Methods

### 4.1. Chemical and Reagents

Methanol, Acetonitrile, and Formic Acid HPLC grade were acquired from Merck (Darmstadt, Germany). Ultrapure water was used throughout and was produced by a Milli-Q Ultrapure water system with a resistivity of 18.2 MΩ.cm at 25 °C (Millipore, Bedford, MA, USA). For the NMR experiments, methanol-*d*_4_ (>99.8 atom % D) and deuterium oxide (>99.8 atom % D) were purchased from Sigma-Aldrich USA (St. Louis, MO, USA). Dimethyl sulfoxide (DMSO) was purchased from Panreac AppliChem. RPMI-1640 and DMEM medium, fetal bovine serum (FBS), and penicillin-streptomycin were obtained from Gibco BRL (Grand Island, NY, USA). Chemical standards, namely, Gallic acid (purity: >98%), Chlorogenic acid (purity: >98%), Caffeic acid (purity: >98%), Isoorientin (purity: >98%), Ampelopsin (purity: >98%), Rutin (purity: >98%), Vitexin (purity: >98%), *p*-Coumaric acid (purity: >98%), Sinapic acid (purity: >98%), Morin (purity: >98%), Coumarin (purity: >98%), Quercetin (purity: >98%), Cinnamic acid (purity: >98%), and Naringenin (purity: >98%) were obtained from Sigma-Aldrich (St. Louis, MO, USA).

### 4.2. Plant Material, Extraction

Leaves of *Guadua incana* Londoño were collected from the bamboo collection of El Bambusal Farm, Quindío, Colombia (GPS 4°31′15″ N, 75°48′0″ W) and identified by Néstor García and Ximena Londoño at the Herbarium of the Pontificia Universidad Javeriana (HPUJ) and were deposited with voucher specimen number HPUJ-30723. The collection was performed under the Contract for Access to Genetic Resources and Derived Products (Contract No. 212/RGE 0287-6) granted by the Ministerio de Medioambiente y Desarrollo Sostenible to the Pontificia Universidad Javeriana (PUJ) and the Colombia Científica/GAT Program. The plant material was dried in an oven with circulating air at 40 °C for 96 h (about 4 days) for subsequent grinding. After grinding, extraction of the dried and ground plant material was carried out through percolation with ethanol (96% EtOH) at a ratio of 1:10 (*w*/*v*) at room temperature. The extraction process was conducted in 4 cycles of 24 h each. The bamboo leaf extract obtained from *G. incana* (BLEGI) in the different cycles was combined and concentrated under reduced pressure at a temperature not exceeding 40 °C using a rotary evaporator. The BLEGI extract was purified using a C18 cartridge to remove nonpolar interferences, such as lipids, fats, and other hydrophobic residues, aiming to obtain an extract enriched mainly in flavonoids and other compounds for subsequent biological assays and metabolomic analyses.

### 4.3. Chemical Characterization of the BLEGI

#### 4.3.1. Chemical Analysis through LC-MS

The samples were analyzed using a Waters ACQUITY UPLC high-resolution system in conjunction with a Waters Xevo G2-S QTOF mass spectrometer (Waters Corporation, Milford, MA, USA). A total of 2 µL of the sample was injected into a Waters ACQUITY UPLC HSS T3 C18 (2.1 × 100 mm, 1.8 μm) column (Waters Corporation, Wexford, Ireland), maintaining the temperature at 30 °C. The separation was carried out using two mobile phases, eluent A (water with 0.1% formic acid) and eluent B (acetonitrile with 0.1% formic acid), at a flow rate of 0.5 mL/min. The elution gradient was programmed as follows: 10–100% B from 0 to 10 min, 100% B from 10 to 14 min, 100–5% B from 14 to 15 min, and 5% B from 15 to 20 min. Mass spectrometry was performed on a Xevo G2-S QTOF instrument using electrospray ionization (ESI). Mass spectral data were acquired in negative (ESI^−^) and positive (ESI^+^) ionization modes over a mass range of *m*/*z* 100–1500 Da using dependent data acquisition (DDA) mode at a scan rate set at 0.1 s, followed by MS/MS scanning of the most intense ions. For MS/MS fragmentation, a low-collision-energy ramp varying between 10 and 40 eV as well as a high-collision-energy ramp varying between 50 and 80 eV were applied to acquire the data in centroid format. The QTOF instrument operated at a frequency of 6.0 GHz and 76.0 µs, using high resolution calibrated with the reference mass correction for Leucine-enkephalin (C_28_H_37_N_5_O_7_) as a lock mass compound in positive ion mode ([M + H]^+^ = 556.2771) and negative ion mode ([M − H]^−^ = 554.2615), respectively. The spectrometer parameters were as follows: capillary voltage was set to 2.2 kV (ESI−) or 2.6 kV (ESI+), cone voltage was set to 40 V, source temperature was maintained at 120 °C, cone gas was set to 50 L/h, and desolvation gas was set to 800 L/h at 300 °C. Retention time data LC-MS analyzes of the chemical standards are detailed in Table S3.

#### 4.3.2. GNPS Classical Molecular MS/MS Network Analyses

The MS/MS data files in mzML format were submitted to the GNPS online platform (http://gnps.ucsd.edu) accessed on 3 May 2024. The molecular networking of BLEGI was obtained using parameters provided in Table S4 and described by Aron et al., 2020 [61]. The attribute table of the generated nodes was visualized in Cytoscape version 3.7.2 software to analyze the molecular network.

### 4.4. Cell Lines and Culture Conditions

The human colorectal cancer cell line HCT-116 was obtained from the American Type Culture Collection. Cells were routinely cultured in RPMI 1640 medium supplemented with 10% FBS and 1% penicillin-streptomycin. The cells were incubated at 37 °C in a humidified atmosphere with 5% CO_2_. For all assays, HCT-116 cells were cultured and used at 80% confluency.

### 4.5. Cytotoxicity Assay

Before the metabolomics experiment, an assessment of the cytotoxic potential of the BLEGI was conducted using the MTT assay [62]. Briefly, HCT-116 cells were cultivated in 96-well plates at a density of 1 × 10^6^ cells per well in growth medium. Following a 24 h incubation period, the cells were rinsed with a PBS solution and treated with the extract at various concentrations (0.0032 to 50 µg/mL). Respective controls were included on each plate, comprising a blank (medium only), a solvent control (0.1% DMSO), and a positive control (doxorubicin). After a 72 h incubation, the cells underwent another PBS wash and were further maintained for an additional 3 h in medium containing 0.5 mg/mL MTT. The resulting formazan was dissolved in 150 µL of DMSO, and absorbance was measured at 570 nm using a Bio-Tek Synergy HTX multi-mode reader (BioTeck Instruments, Winooski, VT, USA). Two independent experiments were performed in duplicates, and the IC_50_ values and their 95% confidence interval were calculated by sigmoidal nonlinear regression using GraphPad Prism 8 software (San Diego, CA, USA).

### 4.6. Immunofluorescence Analysis

In the immunofluorescence assay, cells were cultured on coverslips in 35 mm Petri dishes at a concentration of 1 × 10^4^ cells/mL. After 24 h, they were treated with a concentration of 1.23 µg/mL of BLEGI, and after the treatment time (72 h), they were fixed in 3.7% formaldehyde for 20 min. Subsequently, they were washed with PBS, permeabilized with 0.5% Triton X-100 for 20 min, and washed again. After permeabilization, the primary antibody (anti-α-tubulin, 1:50, Sigma-Aldrich, St. Louis, MO, USA) was added and left in contact with the cells overnight. Following the incubation period with the primary antibody, the cultures were washed with PBS, and the secondary antibody coupled to fluorescein (Alexa Fluor 488 Conjugate, 1:50, Cell Signaling Technology, Danvers, MA, USA) was added and incubated for 2 h. Subsequently, the nuclei were stained with DAPI (4′,6-diamidino-2-phenylindole) (1:100, Sigma-Aldrich, St. Louis, MO, USA). The slides were then mounted with Vecta-Shield anti-fading (Vector Laboratories, Burlingame, CA, USA). Images were acquired using a LionHeart FX fluorescence microscope (Biotek, Winooski, VT, USA).

### 4.7. Nitric Oxide (NO) Scavenging Assay

The evaluation of the NO scavenger was performed according to a methodology adapted from Marcocci et al. and Bates et al. [63,64]. The assay is based on the ability of sodium nitroprusside (NPS) molecules to release NO spontaneously in PBS buffer when under the influence of light. NO, in turn, is transformed into nitrite (NO_2_^−^) when reacting with molecular oxygen in the medium and can, therefore, be quantified by adding Griess reagent (1% *w*/*v* sulfanilamide, 0.1% *w*/*v* of naphthyl ethylenediamine and 2.5% *v*/*v* of ortho-phosphoric acid) to form a pink chromophore with high absorption at 540 nm. To this end, NPS (1.25 mM) was prepared in phosphate buffer (0.1 M and pH 7.0) in the absence of light. In a 96-well plate, 50 μL of NPS and 50 μL of samples at different concentrations (25, 50, 100, and 200 μg/mL) were added and incubated for 40 min at room temperature with strong exposure to UV light. After incubation, 100 μL of Griess reagent was added and the reaction mixture was read at 540 nm. A calibration curve was also performed with sodium nitrite (NaNO_2_) to represent the data as concentration of NO_2_^−^ formed (Figure S6). Quercetin was used as an inhibition standard.

### 4.8. Macrophages Cell Culture and Viability

The murine macrophage cell line RAW 264.7 (ATCC TIB 71) was provided by the cell bank of Rio de Janeiro, Brazil. The cells were maintained in DMEM high glucose medium (Dulbecco’s modified eagle medium) supplemented with 10% FBS (*v*/*v*) and incubated in an oven at 37 °C with an atmosphere containing 5% CO_2_ at different periods until reaching a confluence of approximately 70 to 90%. Afterward, the medium was removed and the cells were washed 3 times with PBS and then removed with the aid of a cell scraper and finally counted in a Neubauer chamber to obtain cell concentration values. A total of 100 µL of medium containing approximately 50 × 10^4^ cells/mL was distributed in microplates (96 wells) incubated under the same conditions as above. After 24 h, the medium was removed, the wells were washed with PBS 3 times, and each well was treated with 3 concentrations of each sample (50 to 200 μg/mL) in triplicate. After incubation for 24 h, cell viability was determined by the MTT test [62]. The results were presented as mean ± standard deviation.

### 4.9. Inhibition of NO Produced by Macrophages

For NO assays, murine macrophage cultures RAW 264.7 (ATCC TIB-71) were used. Briefly, the cells were distributed in 96-well microplates at a concentration of 5 × 10^5^ viable cells/well/mL in complete DMEM medium and incubated for 2 h (37 °C with 5% CO_2_) for cell adhesion. After this period, the supernatant was discarded, and the cells were washed with sterile PBS. They were then stimulated with *Escherichia coli* Lipopolysaccharide (LPS) at a concentration of 1 µg/mL, receiving, at the same time, different concentrations of samples (2 and 200 μg/mL) in supplemented DMEM, these being the concentrations previously determined by the cytotoxicity assay. The plates were incubated overnight (37 °C with 5% CO_2_). After this period, culture supernatants were collected for detection and quantification of NO using the Griess method [65]. Data were presented in NO_2_^−^ concentration (mM), using a standard curve for NaNO_2_ (Figure S7). N(ω)-nitro-l-arginine methyl ester (L-NAME) was used as an inhibition control.

### 4.10. Statistical Analysis

All results are represented as the mean ± standard deviation (SD) of triplicate data. Differences observed between the concentrations were achieved by one-way analysis of variance (ANOVA) followed by Tukey’s post hoc test and values of * *p*-value < 0.05 and ** *p*-value < 0.01 were considered significant. Statistical analyses were performed using GraphPad Prism 8 software (San Diego, CA, USA).

### 4.11. Untargeted Metabolomic Analysis

#### 4.11.1. Cell Treatment

The cells were uniformly cultured in a T75 cell culture flask under the same conditions. Ten replicates of both the BLEGI-treated group and the control group (untreated cells) were prepared for the metabolomic study. Additionally, a medium blank (process blank) without cells was placed on each plate. The concentration selected for treatment of the cells with BLEGI corresponded to the calculated IC_50_ value. Cells were then seeded at a density of 3 × 10^6^ cells/well in 6-well cell culture plates using 5 mL of supplemented medium. Afterward, the plates were incubated for 24 h to facilitate cell adhesion. Once the incubation was completed, fresh medium containing the concentration of BLEGI was added for cell treatment. In all cases, the final concentration of DMSO in the medium did not exceed 0.01%. A control group of cells subjected to the same conditions was used. After a 72 h incubation, the sampling process was carried out.

#### 4.11.2. Metabolite Extraction

First, the culture medium was removed, and the cells were quickly washed with 1 mL of PBS. Cell quenching was then performed by placing the plates in an ice bucket, and 1 mL of methanol cooled to −80 °C was added to each well and left for a period of 10 min. Subsequently, the cells (endometabolome) were carefully scraped from the plates, and two aliquots of the solution were stored in 2 mL Eppendorf tubes on dry ice for subsequent cell lysis by thermal shock in liquid nitrogen and sonication for 5 min each (this procedure was repeated three times). Finally, two aliquots were stored from the removed culture medium (exometabolome). The endo- and exometabolome samples were centrifuged at 15,000 rpm for 10 min at 4 °C to remove suspended particles. The samples were then freeze-dried and stored at −80 °C until metabolomic analysis.

#### 4.11.3. Sample Preparation for Metabolomic Analysis

For the analysis of endo- and exometabolome by LC-MS, the lyophilizate obtained above was resuspended in 500 µL of MeOH cooled to −20 °C, while for the analysis by ^1^H-NMR, the previously obtained lyophilizate was resuspended in 100 µL of methanol-*d*_4_ (CD_3_OD) and 100 µL of phosphate buffer (KH_2_PO_4_) in deuterium oxide (D_2_O) (pH 6.0) Isotope with 99.8% purity, the solution was sonicated (frequency of 50 kHz/20 min) and centrifugated (10,000 rpm/5 min), and placed in 3 mm NMR tubes for further 1D NMR analyses. Multiple QC samples were prepared by pooling and mixing identical volumes of each extract sample. This was done to verify system performance and ensure reproducibility in sample analysis. To evaluate instrument consistency, injections of the pooled QC samples were performed after every four sample injections.

#### 4.11.4. LC-MS Metabolomic Profiling Analysis

LC-MS analysis of the endo- and exometabolome samples obtained from HCT-116 cells was performed under the same conditions described in Section 4.3.1.

#### 4.11.5. ^1^H-NMR Fingerprinting Analysis

^1^H-NMR spectra were recorded at 25 °C on a Bruker Avance III HD 600 MHz spectrometer (Bruker, Karlsruhe, Germany) operating at a proton NMR frequency of 600.13 MHz. Each ^1^H-NMR spectrum comprised 128 scans with the following parameters: 0.16 Hz/point, pulse width (PW) = 30 (11.3 μs), and relaxation delay (RD) = 1.5 s. Free induction decays (FIDs) underwent Fourier transformation with a line broadening (LB) of 0.3 Hz. Deuterated methanol was used as the internal lock. Chemical shifts were denoted in δ (ppm), and coupling constants were expressed in hertz.

#### 4.11.6. Data Processing and Statistical Analysis

For the processing of the data obtained from LC-MS, the analysis was carried out using MZmine v2.53 software to perform a deconvolution, alignment, and integration process. For the processing of the data obtained from ^1^H-NMR, the analysis was carried out using NMRProcFlow v1.4 online software, in which a process of alignment and normalization of the data was performed. Additionally, the data were segmented and reduced using the clustering method with a window of 0.04 ppm. SIMCA 18.0 (Umetrics, Umea, Sweden) was used to perform multivariate analyses (MVA). MVA was used to build models such as PCA and OPLS-DA in relation to the VIP. The variables presenting statistical significance were determined by considering a *p*-value corrected with FDR of less than 0.05, and a VIP greater than 1. This confirmation was based on the OPLS-DA models together with a significance assessment by means of CV-ANOVA with a value of less than 0.05. To establish *p*-valued characteristics, univariate analysis (UVA) was performed using MetaboAnalyst 6.0 server [66,67].

#### 4.11.7. Metabolites Identification

Differential annotation of metabolites was carried out considering mass accuracy with a maximum error of 10 ppm, as well as isotopic pattern distribution and adduct formation. For this purpose, several online public databases, such as METLIN (http://metlin.scripps.edu) accesed on 22 November 2023, KEGG (http://genome.jp/kegg) accesed on 22 November 2023, HMDB (https://hmdb.ca/) accesed on 22 November 2023, PubChem (https://pubchem.ncbi.nlm.nih.gov/) accesed on 22 November 2023, and ChEBI (https://www.ebi.ac.uk/chebi/) accesed on 22 November 2023, were used through the CEU mass mediation tool. Metabolite identification was based on the comparison between the obtained experimental MS/MS spectra and the predicted spectra present in the databases and other tools described in the literature. In addition to the chemical characterization process, we also utilized the GNPS web platform (https://gnps.ucsd.edu/ProteoSAFe/static/gnps-splash.jsp) accessed on 3 May 2024 and manual interpretation with MassLynx software version 4.01 (Waters Company, Milford, MA, USA). Furthermore, an in-house library was developed using metabolites previously isolated and reported from the bamboo species subfamily (Subfamily: Bambusoideae) within the research group, which facilitated the confirmation of metabolite identities.

#### 4.11.8. Mapping of Altered Metabolic Pathways

The analysis of the metabolic pathways affected in the treated cells was conducted using the “Pathway Analysis” tool of the MetaboAnalyst 6.0 server (http://www.metaboanalyst.ca/) accesed on 14 February 2024. For this purpose, the altered compounds were annotated and compared with the *Homo sapiens* metabolome (KEGG) available on the same server.

## 5. Conclusions

To the best of our knowledge, this is the first study to investigate possible alterations in metabolites associated with BLEGI-treated HCT-116 colon cancer cells. In this study, endo- and exometabolomic profiles were used to assess metabolite changes and identify altered metabolic pathways, proposing a possible mechanism of action. The tentatively identified metabolites suggest the possibility that the altered pathways are primarily associated with glutathione metabolism. Metabolomic analysis revealed that BLEGI may inhibit tumor progression, cause damage to tumor cells, and stimulate apoptosis. Furthermore, the identification of reduced levels of sphingosine and glutathione, along with increased levels of glutathione disulfide, potentially allows the activation of apoptosis signaling pathways, which may be one of the proposed mechanisms of action mediated by the induction of oxidative stress. However, more in-depth studies are needed to confirm these findings of altered metabolic pathways and the mechanisms of action through which the extract exerts its therapeutic effect. Moreover, the anti-inflammatory potential of BLEGI was demonstrated in the inhibition of nitric oxide in LPS-stimulated macrophage cells. Our results globally demonstrate the effect of BLEGI at the metabolic level in colon cancer lines, providing clues for the identification of new therapeutic targets in the treatment of this disease.

## Data Availability

Data are contained within the article and Supplementary Materials.

## Supplementary Materials

The following supporting information can be downloaded at: https://www.mdpi.com/article/10.3390/molecules29132985/s1, Figure S1: MS/MS spectrum match feature of GNPS showing the similarity of fragments patterns of the experimental and library data; Figure S2: PCA score plot including quality controls (QCs) and all samples from LC-MS and ^1^H-NMR analysis; Figure S3: Cross-validation plots of the OPLS-DA models with 100 permutation tests. BLEGI-treated group (*n* = 10) and untreated group (*n* = 10); Figure S4: Volcano plot of metabolites from the (A) endometabolome and (B) exometabolome that met the *p*-value and fold change criteria, comparing the group of cells treated with BLEGI versus untreated cells; Figure S5: Representative ^1^H-NMR spectra of the (A) endometabolome and (B) exometabolome at 72 h of BLEGI-treated cells; Table S1: List of all metabolites identified in the endometabolome of cancer cells HCT-116 treated with BLEGI; Table S2: List of all metabolites identified in the exometabolome of cancer cells HCT-116 treated with BLEGI; Table S3: Retention times of chemical standards in the LC-MS analyses; Table S4: Parameters used in the GNPS Classical Molecular Networking; Figure S6: Calibration curve with sodium nitrite (NaNO2) to represent the data in concentration of chemical NO2- formed; Figure S7: Calibration curve with sodium nitrite (NaNO_2_) to represent the data in concentration of cellular NO_2_^−^ formed.
