# Supplementary material for: Uncovering Metabolic Alterations in HCT-116 Colon Cancer Cells upon Exposure to Bamboo Leaf Extract Obtained from Guadua incana Londoño
Source: Molecules. 2024 Jun 23;29(13):2985. doi: 10.3390/molecules29132985 (PMC11243423; doi:10.3390/molecules29132985)
Supplement: Supplementary file 1 [file molecules-29-02985-s001.zip › molecules-3024362-supplementary.pdf]

Supplementary Materials

Uncovering Metabolic Alterations in HCT-116 Colon Cancer Cells upon Exposure to Bamboo Leaf Extract obtained from *Guadua incana* Londoño

Table of Contents:

Figure S1. MS/MS spectrum match feature of GNPS showing the similarity of fragments patterns of the experimental and library data.

Figure S2. PCA score plot including quality controls (QCs) and all samples from LC-MS and ^{1}H-NMR analysis.

Figure S3. Cross-validation plots of the OPLS-DA models with 100 permutation tests. BLEGI-treated group (n = 10) and untreated group (n = 10).

Figure S4. Volcano plot of metabolites from the A) endometabolome and B) exometabolome that met the p-value and fold change criteria, comparing the group of cells treated with BLEGI versus untreated cells.

Figure S5. Representative ^{1}H-NMR spectra of the A) endometabolome and B) exometabolome at 72 h of BLEGI-treated cells.

Table S1. List of all metabolites identified in the endometabolome of cancer cells HCT-116 treated with BLEGI.

Table S2. List of all metabolites identified in the exometabolome of cancer cells HCT-116 treated with BLEGI.

Table S3. Retention times of chemical standards in the LC-MS analyses**.**

Table S4. Parameters used in the GNPS Classical Molecular Networking.

Figure S6. Calibration curve with sodium nitrite ($NaNO_2$) to represent the data in concentration of chemical NO_2^- formed.

Figure S7. Calibration curve with sodium nitrite ($NaNO_2$) to represent the data in concentration of cellular NO_2^- formed.

References

Compound 19

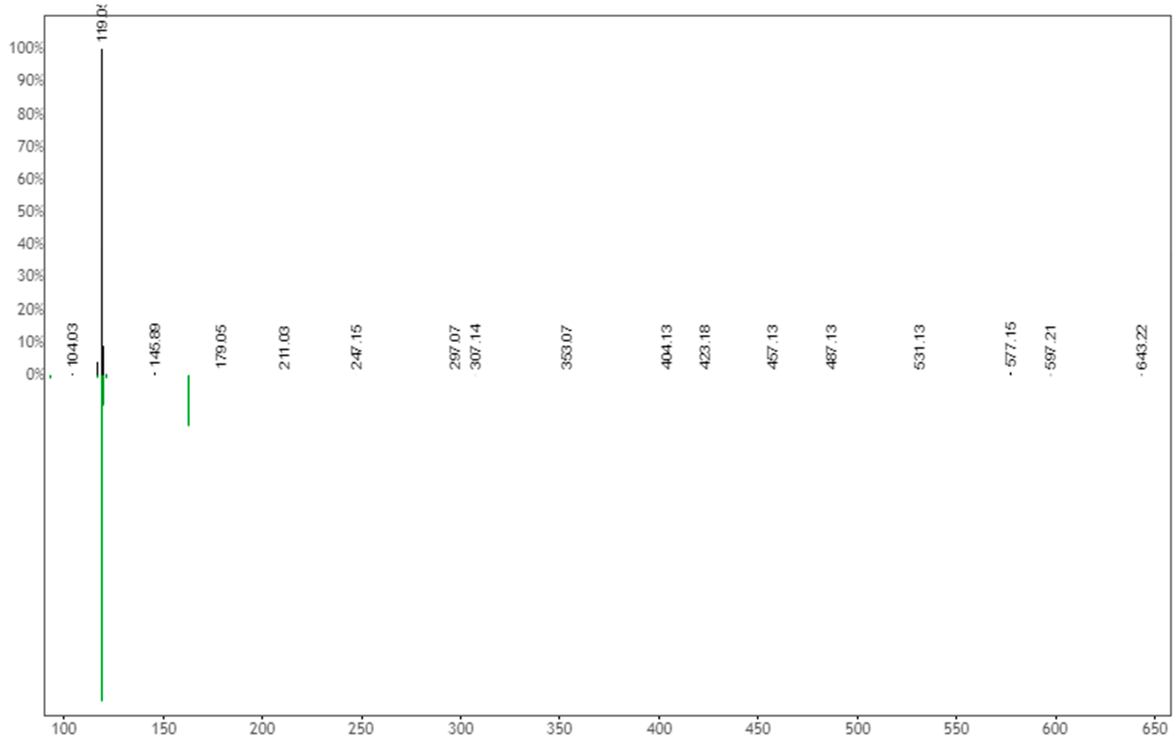

Compound 3

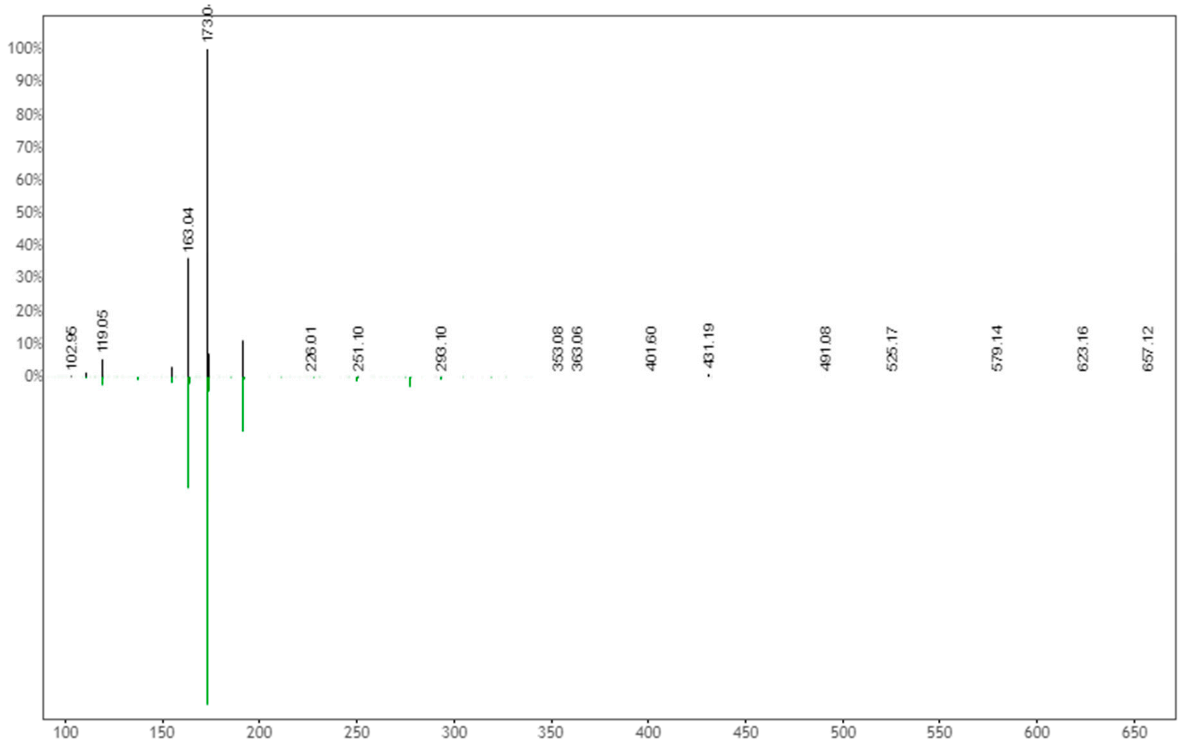

Compound 6

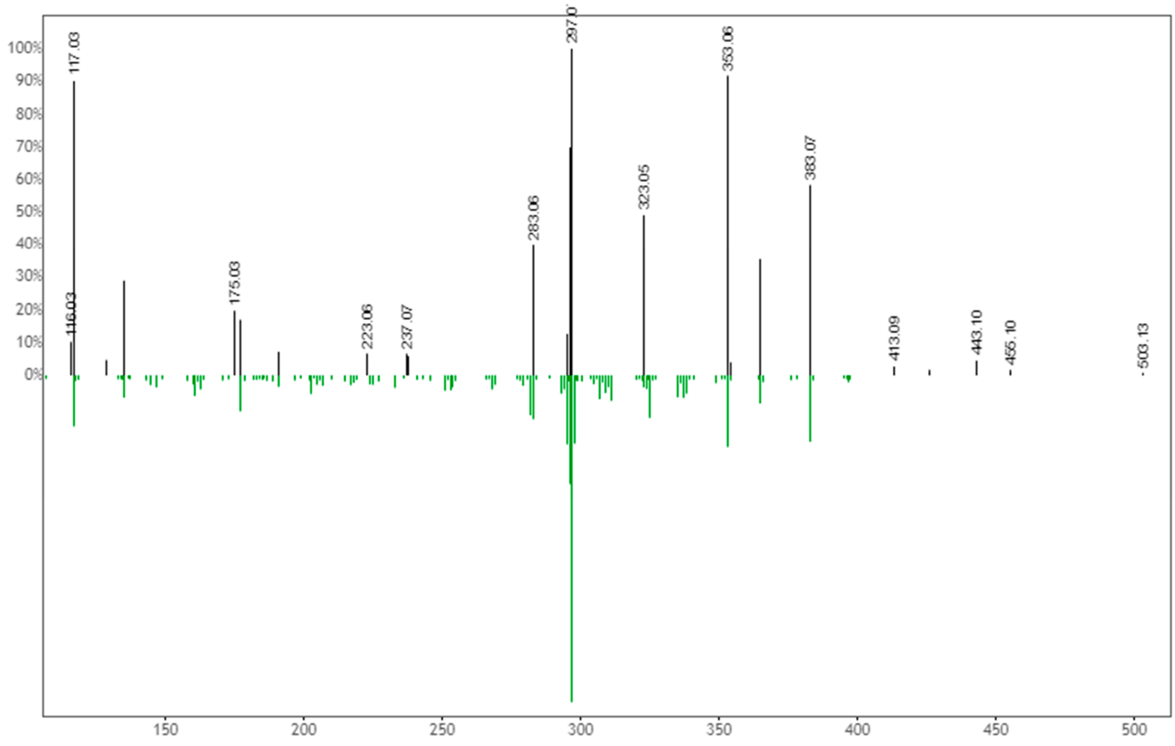

Compound 8

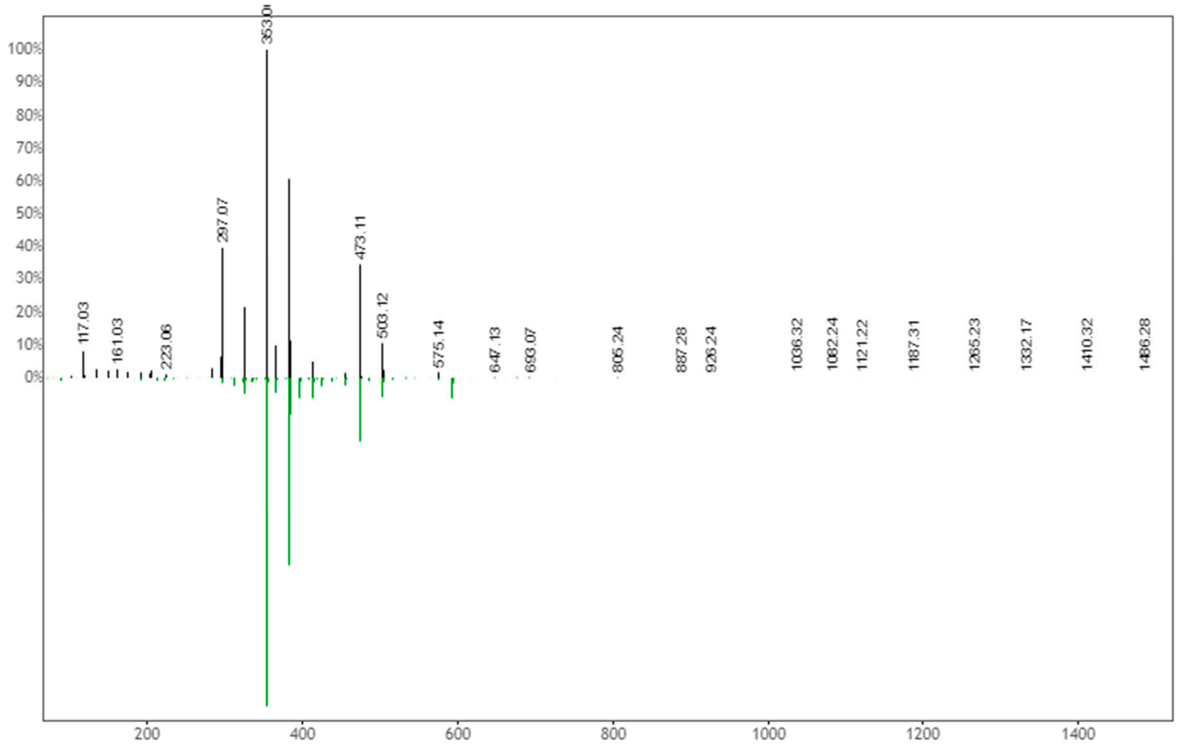

Compound 13

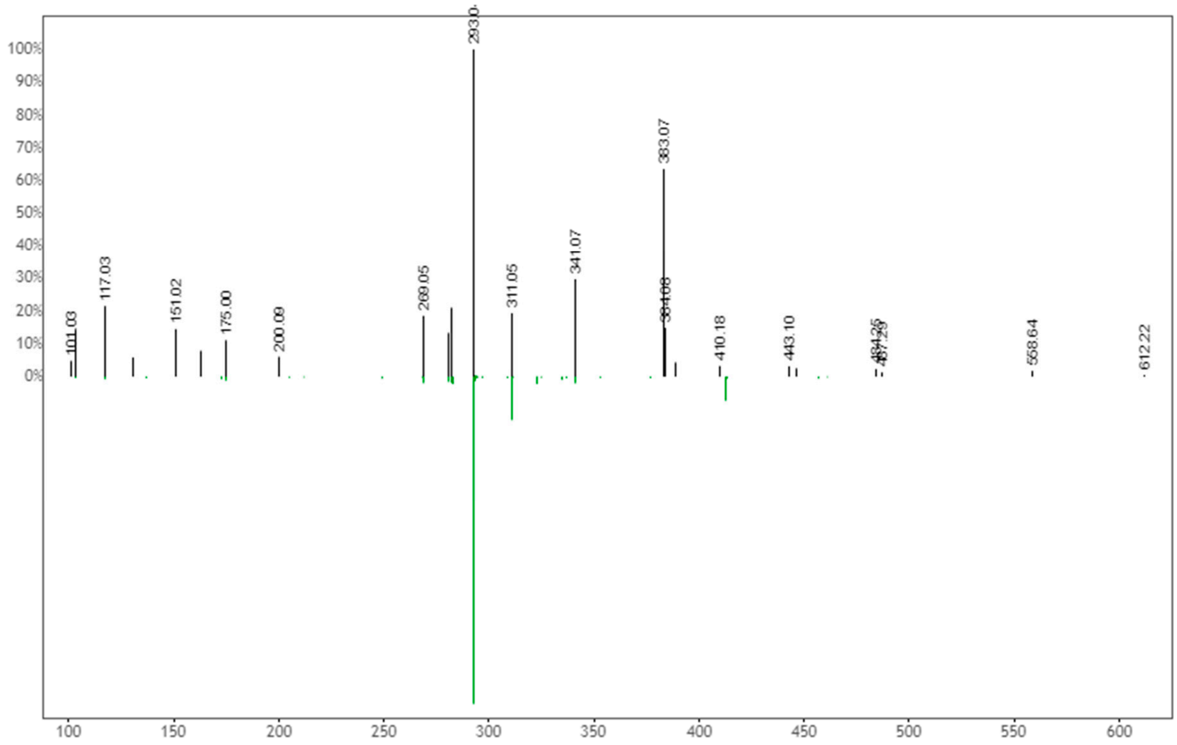

Compound 14

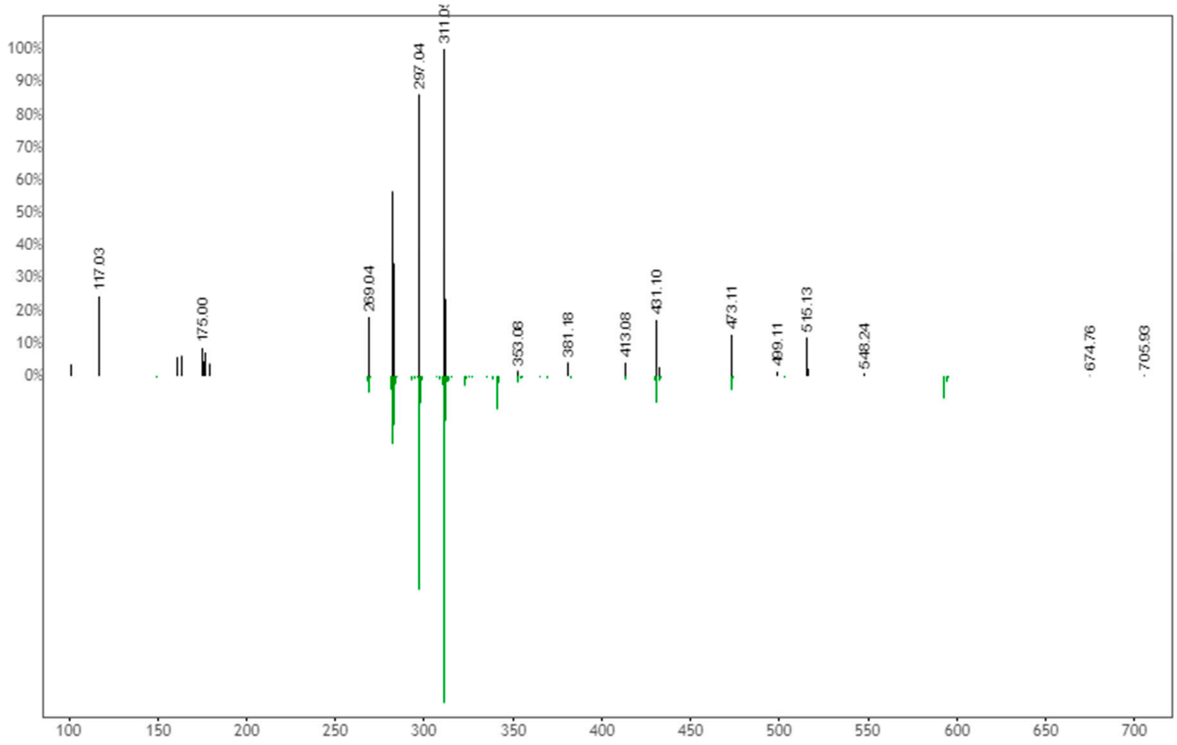

Compound 15

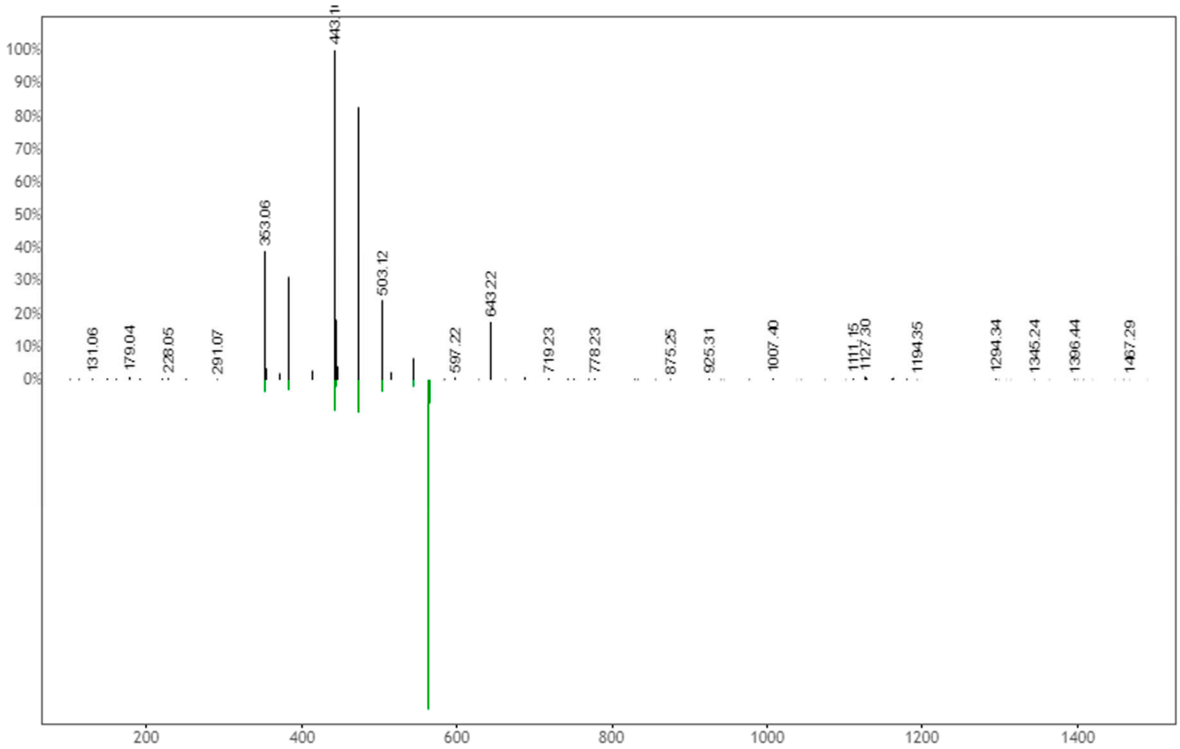

Compound 21

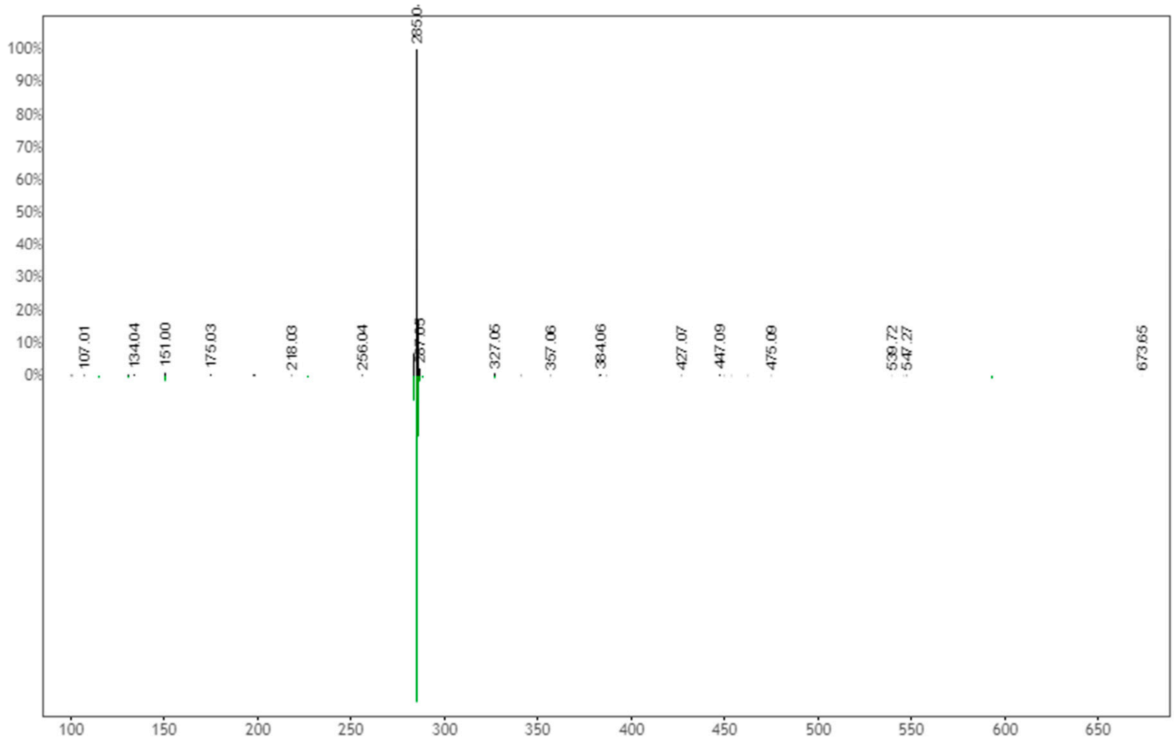

Compound 26

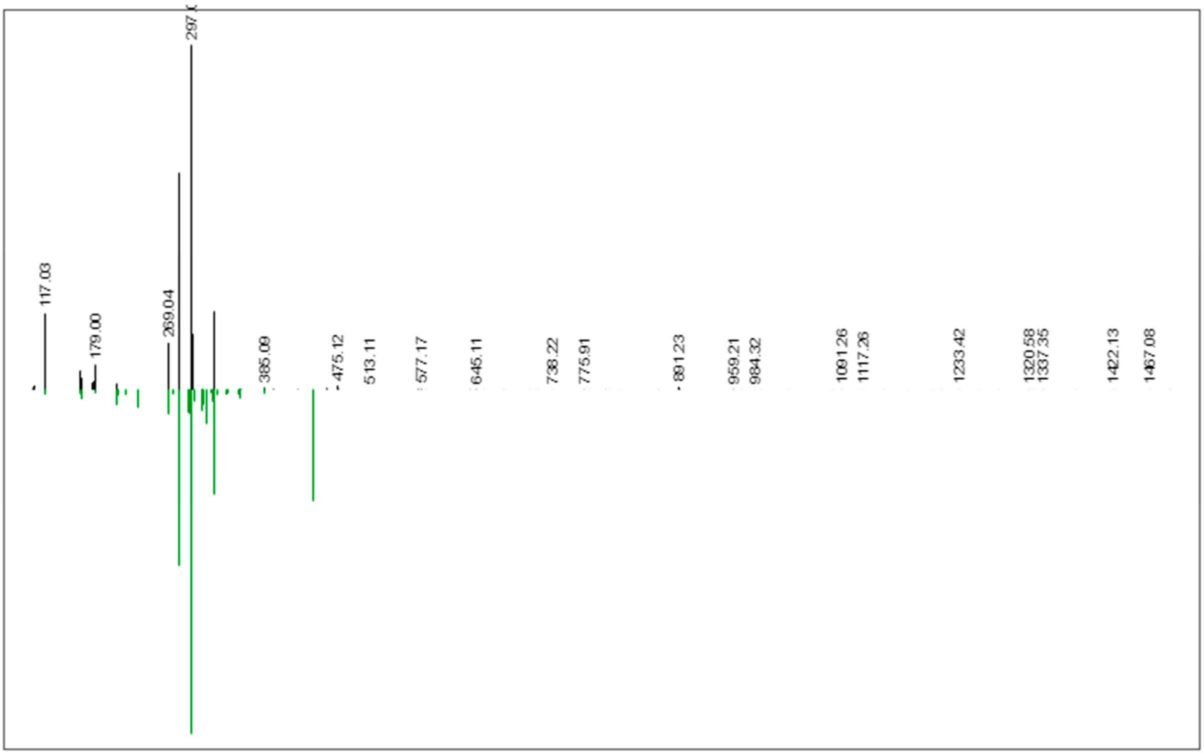

Figure S1. MS/MS spectrum match feature of GNPS showing the similarity of fragments patterns of the experimental and library data.

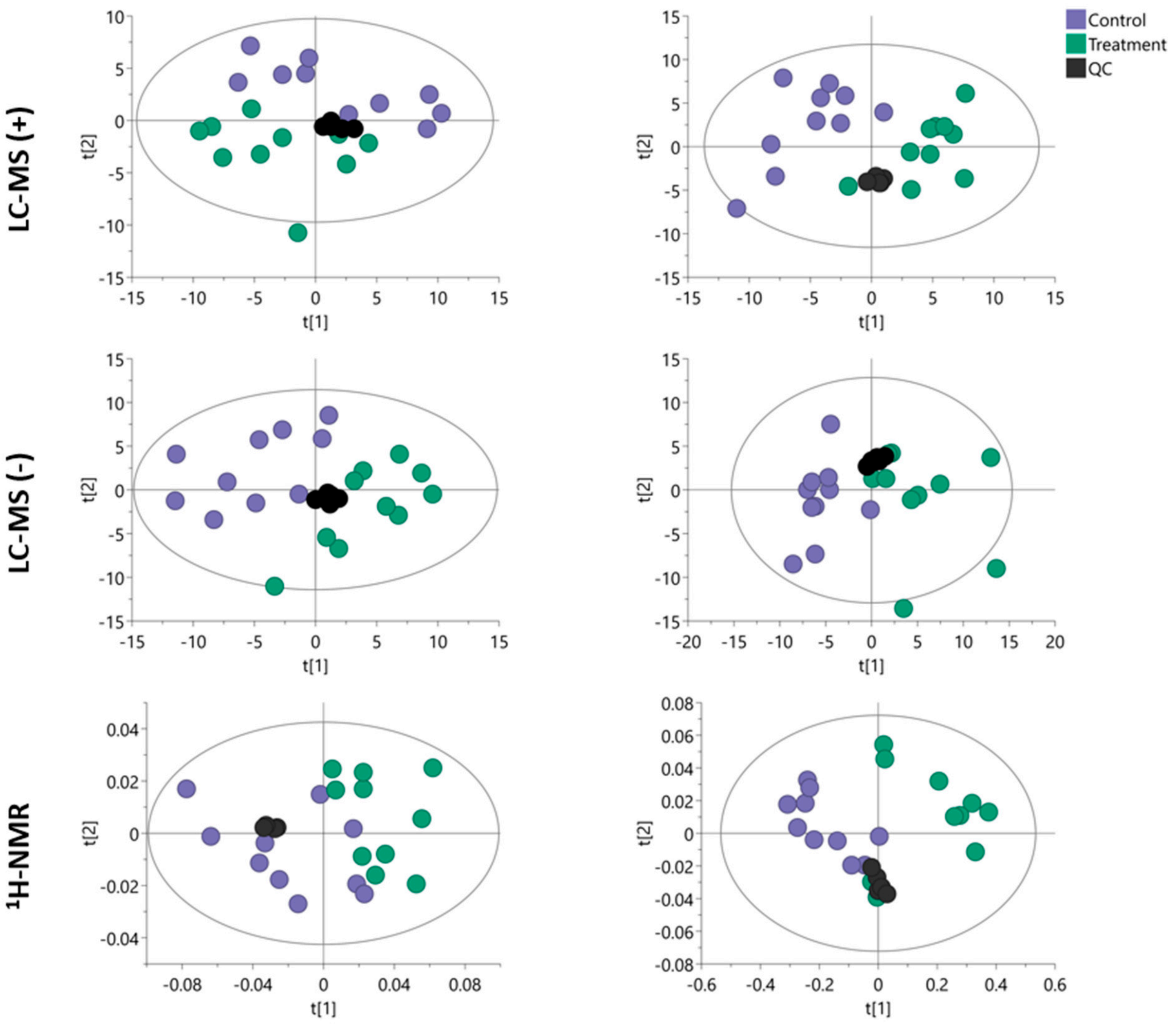

Figure S2. PCA score plot including quality controls (QCs) and all samples from LC-MS and ^{1}H-NMR analysis.

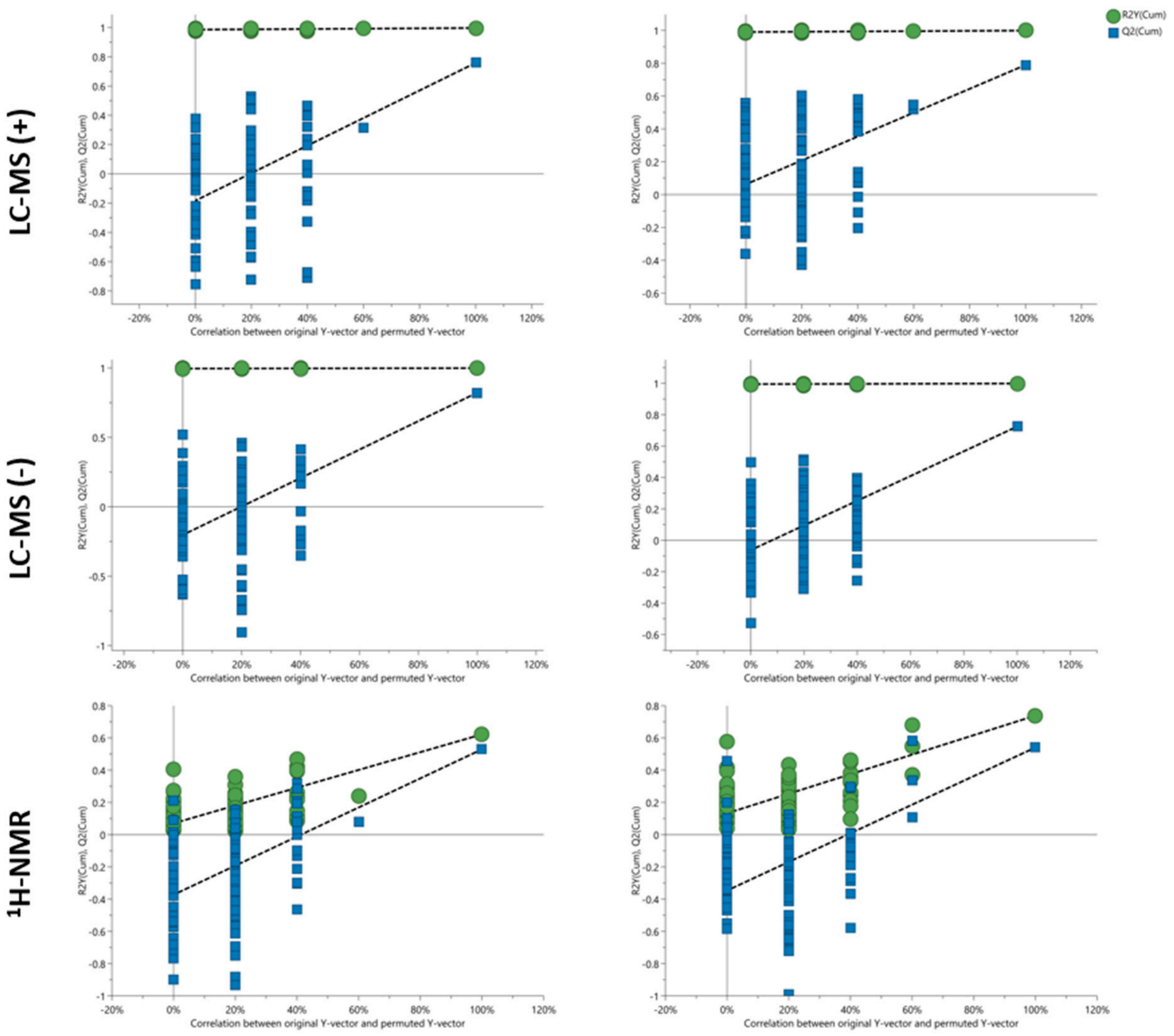

Figure S3. Cross-validation plots of the OPLS-DA models with 100 permutation tests. BLEGI-treated group (n = 10) and untreated group (n = 10).

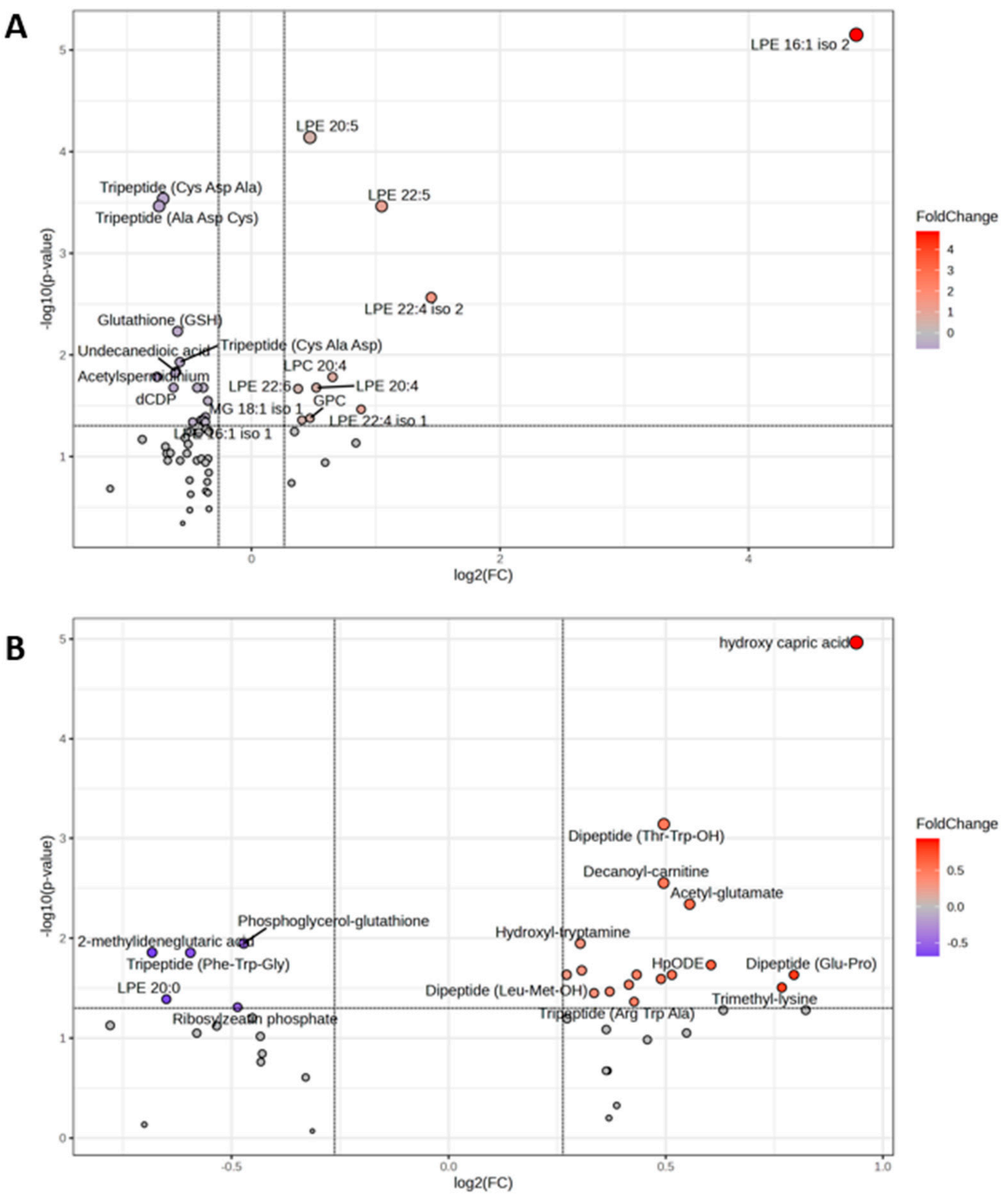

Figure S4. Volcano plot of metabolites from the A) endometabolome and B) exometabolome that met the *p*-value and fold change criteria, comparing the group of cells treated with BLEGI versus untreated cells.

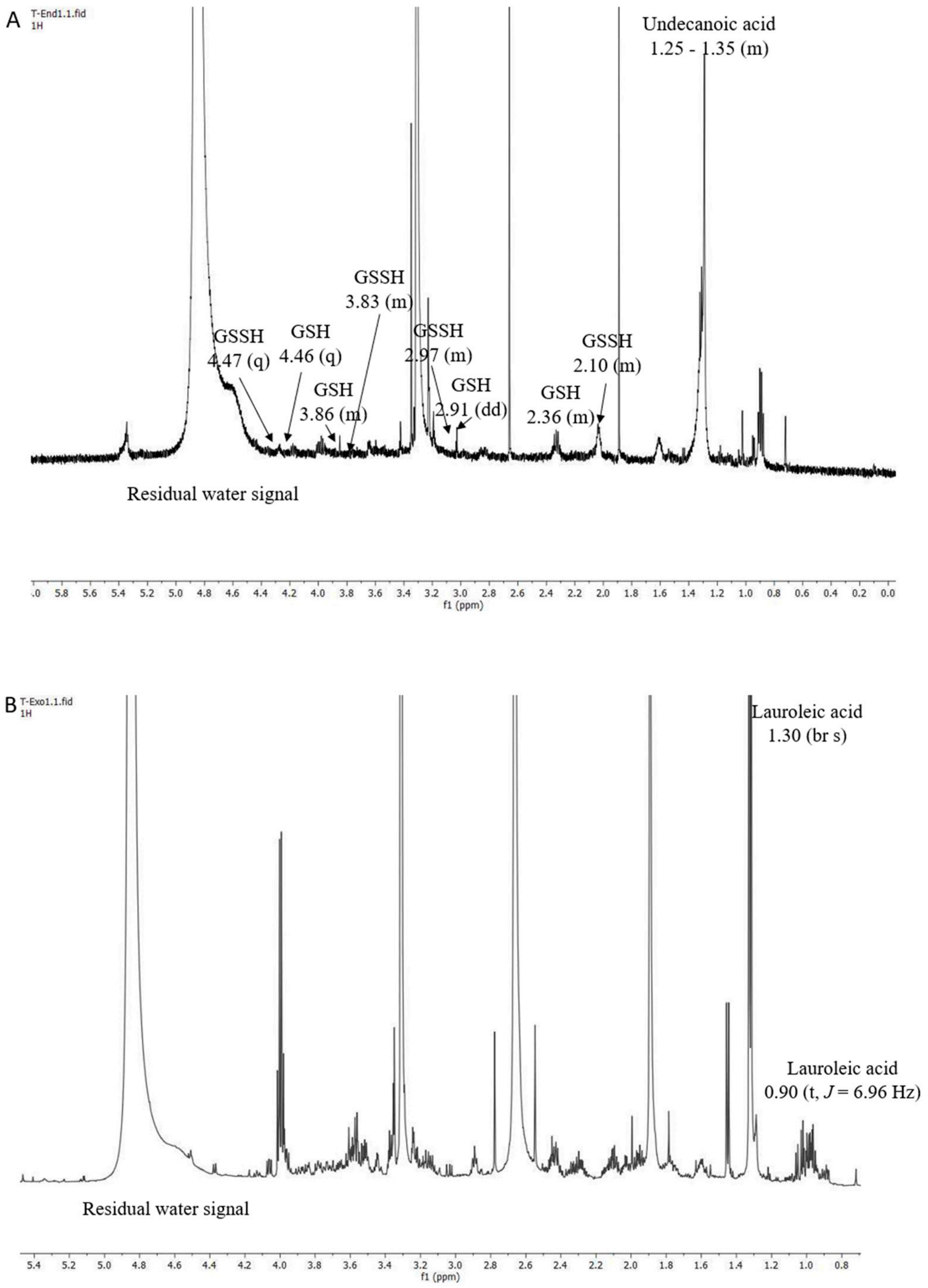

Figure S5. Representative ^{1}H-NMR spectra of the A) endometabolome and B) exometabolome at 72 h of BLEGI-treated cells. The experimental data were compared with reports in the literature based on chemical shifts, multiplicities, and coupling constants for each metabolite, assigning the most representative signal for each molecule.

Table S1. List of all metabolites identified in the endometabolome of cancer cells HCT-116 treated with BLEGI.

Compounds	Formula	Mass	RT (min)	Error (ppm)	Adduct	δ ^{1}H (ppm) and Multiplicity, *J* in Hz	Assignments	[a]CV for QC (%)	Analytical platform	DET	[b]ID Level	BLEGI-treated cells vs. untreated cells		
												[c]Fold Change	[d]*p* value*	[e]VIP
Organic acids and derivatives														
Acetylspermidinium	$C_9H_{21}N_3O$	187.1685	1.10	1	$[M+H]^+$	-	-	9.45	LC-QTOF-MS	ESI +	3	0.65	2.65×10^{-3}	1.64
Glutathione (GSH)	$C_{10}H_{17}N_3O_6S$	307.0838	1.13	3	$[M+H]^+$	2.91 (dd); 2.36 (m) 4.46 (q); 3.86 (m)	-	9.65	LC-QTOF-MS / ^{1}H-NMR	ESI + / ^{1}H	2	0.67	2.87×10^{-2}*	1.61
Glutathione disulfide (GSSG)	$C_{20}H_{32}N_6O_{12}S_2$	612.1520	8.54	1	$[M-H]^-$	2.10 (m); 2.97 (m) 4.47 (q); 3.83 (m)	-	3.77	LC-QTOF-MS / ^{1}H-NMR	ESI - / ^{1}H	3	1.32	1.77×10^{-2}	1.24
Glutathione palmitamide	$C_{26}H_{47}N_3O_7S$	545.3135	8.18	0	$[M-H-H_2O]^-$	-	-	3.73	LC-QTOF-MS	ESI -	3	0.79	2.97×10^{-2}	1.05
Carnitines														
Hexadec-2-enoyl carnitine	$C_{23}H_{43}NO_4$	397.3192	8.86	1	$[M+H]^+$	-	-	14.53	LC-QTOF-MS	ESI +	2	0.54	3.93×10^{-2}	1.26
Elaidic carnitine	$C_{25}H_{47}NO_4$	425.3505	9.34	1	$[M+H]^+$	-	-	10.13	LC-QTOF-MS	ESI +	2	0.76	1.84×10^{-2}	1.37
Stearoyl carnitine	$C_{25}H_{49}NO_4$	427.3662	9.88	1	$[M+H]^+$	-	-	9.62	LC-QTOF-MS	ESI +	2	0.77	5.04×10^{-3}	1.50
Arachidyl carnitine	$C_{27}H_{53}NO_4$	455.3975	7.96	1	$[M+H]^+$	-	-	10.37	LC-QTOF-MS	ESI +	2	0.74	5.61×10^{-3}	1.49
Tetradecanoyl carnitine	$C_{21}H_{41}NO_4$	371.3036	3.3	1	$[M+H]^+$	-	-	10.40	LC-QTOF-MS	ESI +	2	0.62	-	1.40
2-Methylbutyroylcarnitine	$C_{12}H_{23}NO_4$	245.1627	9.11	3	$[M+H]^+$	-	-	7.23	LC-QTOF-MS	ESI +	2	0.62	-	1.36
3-hydroxypentadecanoyl carnitine	$C_{22}H_{43}NO_5$	401.3141	1.14	4	$[M+Cl]^-$	-	-	4.18	LC-QTOF-MS	ESI -	2	0.71	2.06×10^{-2}	1.38
Fatty acids														
Methyl arachidonoyl amine	$C_{21}H_{35}NO$	317.2719	6.89	2	$[M+H]^+$	-	-	10.92	LC-QTOF-MS	ESI +	3	0.76	-	1.10
Undecanedioic acid	$C_{11}H_{20}O_4$	216.1362	5.77	8	$[M-H]^-$	1.25 - 1.35 (m)	Aliphatic carbons	10.21	LC-QTOF-MS / ^{1}H-NMR	ESI - / ^{1}H	3	0.66	2.32×10^{-3}	1.46
Glycerophospholipids														
LPC 14:0	$C_{22}H_{46}NO_7P$	467.3012	7.67	1	$[M+H]^+$	-	-	12.13	LC-QTOF-MS	ESI +	2	0.75	-	1.18
LPC 15:0	$C_{23}H_{48}NO_7P$	481.3168	9.37	2	$[M-H-H_2O]^-$	-	-	5.73	LC-QTOF-MS	ESI -	2	0.78	1.43×10^{-2}	1.48
LPC 15:2	$C_{25}H_{52}NO_7P$	509.3481	9.99	3	$[M-H]^-$	-	-	16.24	LC-QTOF-MS	ESI -	2	0.78	-	1.04
LPC 16:0	$C_{24}H_{50}NO_7P$	495.3325	8.86	2	$[M+Cl]^-$	-	-	5.57	LC-QTOF-MS	ESI +	2	1.80	4.39×10^{-2}	1.05
LPC 16:1 iso 1	$C_{24}H_{48}NO_7P$	493.3168	8.17	2	$[M+HCOOH-H]^-$	-	-	4.66	LC-QTOF-MS	ESI +	2	0.73	3.16×10^{-2}	1.13
LPC 16:1 iso 2	$C_{24}H_{48}NO_7P$	493.3168	8.15	2	$[M+HCOOH-H]^-$	-	-	4.03	LC-QTOF-MS	ESI +	2	0.71	-	1.24
LPC 18:1	$C_{26}H_{52}NO_7P$	521.3481	9.11	2	$[M+HCOOH-H]^-$	-	-	4.01	LC-QTOF-MS	ESI +	2	0.79	-	1.06
LPC 20:4	$C_{28}H_{50}NO_7P$	543.3325	8.52	0	$[M+H]^+$	-	-	10.76	LC-QTOF-MS	ESI +	2	1.58	3.50×10^{-3}	1.41

LPC O-18:1	$C_{26}H_{52}NO_7P$	521.3481	9.16	2	$[M+HCOOH-H]^-$	-	-	3.97	LC-QTOF-MS	ESI +	3	0.78	-	1.23
PC 38:4	$C_{46}H_{84}NO_8P$	809.5935	9.69	2	$[M+H]^+$	-	-	10.79	LC-QTOF-MS	ESI +	2	0.69	-	1.25
GPC	$C_8H_{20}NO_6P$	257.1028	0.73	2	$[M+H]^+$	-	-	13.86	LC-QTOF-MS	ESI +	3	1.38	1.57×10^{-2}	1.34
LPE 16:1 iso 1	$C_{21}H_{42}NO_7P$	451.2699	7.93	1	$[M+H]^+$	-	-	9.36	LC-QTOF-MS	ESI +	3	0.78	2.09×10^{-2}	1.34
LPE 16:1 iso 2	$C_{21}H_{42}NO_7P$	451.2699	8.51	4	$[M-H]^-$	-	-	12.49	LC-QTOF-MS	ESI -	2	52.50	2.31×10^{-5}*	1.57
LPE 18:1	$C_{23}H_{46}NO_7P$	479.3012	8.46	5	$[M+Na]^+$	-	-	9.40	LC-QTOF-MS	ESI +	3	1.28	2.74×10^{-2}	1.29
LPE 20:4	$C_{25}H_{44}NO_7P$	501.2855	8.41	2	$[M-H]^-$	-	-	2.68	LC-QTOF-MS	ESI -	3	1.50	5.90×10^{-3}	1.30
LPE 20:5	$C_{25}H_{42}NO_7P$	499.2699	7.97	2	$[M-H]^-$ / $[M+H]^+$	-	-	2.70	LC-QTOF-MS	ESI - / ESI +	2	1.38	2.36×10^{-4}*	1.95
LPE 22:4	$C_{27}H_{48}NO_7P$	529.3168	9.17	5	$[M-H]^-$	-	-	19.63	LC-QTOF-MS	ESI -	3	2.75	7.62×10^{-3}*	1.69
LPE 22:5	$C_{27}H_{46}NO_7P$	527.3012	8.68	1	$[M+H]^+$	-	-	11.35	LC-QTOF-MS	ESI +	2	2.08	3.78×10^{-3}*	1.78
LPE 22:6	$C_{27}H_{44}NO_7P$	525.2855	8.43	1	$[M-H]^-$ / $[M+H]^+$	-	-	2.41	LC-QTOF-MS	ESI - / ESI +	2	1.28	6.42×10^{-3}	1.42
PE 34:0	$C_{39}H_{76}NO_8P$	717.5309	9.62	2	$[M+H]^+$	-	-	13.83	LC-QTOF-MS	ESI +	3	0.69	-	1.17
PE 34:2	$C_{39}H_{74}NO_8P$	715.5152	9.71	1	$[M+H]^+$	-	-	18.47	LC-QTOF-MS	ESI +	3	0.64	-	1.28
PE 36:2	$C_{41}H_{78}NO_8P$	743.5465	9.54	1	$[M-H]^-$	-	-	7.74	LC-QTOF-MS	ESI -	2	0.78	-	1.14
PE P 36:4 iso 1	$C_{41}H_{74}NO_7P$	723.5203	9.64	9	$[M-H]^-$	-	-	15.46	LC-QTOF-MS	ESI -	3	0.70	4.64×10^{-2}	1.03
PE P 36:4 iso 2	$C_{41}H_{74}NO_7P$	723.5203	9.51	10	$[M-H]^-$	-	-	8.21	LC-QTOF-MS	ESI -	3	0.45	-	1.00
LPI 20:3	$C_{29}H_{51}O_{12}P$	622.3118	9.08	2	$[M-H]^-$	-	-	16.18	LC-QTOF-MS	ESI -	2	0.71	-	1.11
PA O-42:6	$C_{45}H_{79}O_7P$	762.5563	9.65	5	$[M-H-H_2O]^-$	-	-	14.08	LC-QTOF-MS	ESI -	3	0.71	-	1.03
PS 37:7	$C_{43}H_{70}NO_{10}P$	791.4737	9.11	3	$[M-H]^-$	-	-	5.52	LC-QTOF-MS	ESI -	3	1.55	-	1.05
PS P 35:0	$C_{41}H_{80}NO_9P$	761.5571	9.64	0	$[M-H-H_2O]^-$	-	-	15.86	LC-QTOF-MS	ESI -	3	1.26	-	1.02
Glycerolipids														
MG 18:1 iso 1	$C_{21}H_{40}O_4$	356.2927	8.90	2	$[M+H-H_2O]^+$	-	-	11.14	LC-QTOF-MS	ESI +	3	0.78	8.75×10^{-3}	1.49
MG 18:1 iso 2	$C_{21}H_{40}O_4$	356.2927	8.89	2	$[M+H-H2O]^+$	-	-	13.46	LC-QTOF-MS	ESI +	3	0.66	-	1.10
Sphingolipids														
Sphingosine	$C_{18}H_{37}NO_2$	299.2824	2.74	2	$[M+H]^+$	-	-	10.13	LC-QTOF-MS	ESI +	3	0.77	2.41×10^{-2}	1.38
SM 34:2	$C_{39}H_{77}N_2O_6P$	700.5519	0.82	1	$[M+H]^+$	-	-	8.89	LC-QTOF-MS	ESI +	3	0.71	-	1.01
Steroids and steroid derivatives														
2-hydroxyethinylestradiol	$C_{20}H_{24}O_3$	312.1725	9.40	6	$[M-H]^-$	-	-	5.28	LC-QTOF-MS	ESI -	3	0.75	-	1.01
Sterol Lipids														
3,12-dihydroxy-24-oxo-7-(sulfoxy)cholan-24-yl]-Glycine	$C_{26}H_{43}NO_9S$	545.2659	0.82	3	$[M+Cl]^-$	-	-	4.17	LC-QTOF-MS	ESI -	3	0.70	2.80×10^{-2}	1.32
Nucleosides, nucleotides, and analogues														

Methylthioadenosine	$C_{11}H_{15}N_5O_3S$	297.0896	1.11	1	$[M+H]^+$	-	-	9.68	LC-QTOF-MS	ESI +	3	0.78	2.53×10^{-2}	1.37
Peptides														
Tripeptide (Cys Ala Asp)	$C_{10}H_{17}N_3O_6S$	307.0838	1.12	2	$[M+H]^+$	-	-	9.44	LC-QTOF-MS	ESI +	3	0.67	1.65×10^{-3}	1.49
Tripeptide (Gly Met Cys)	$C_{10}H_{19}N_3O_4S_2$	309.0817	0.82	1	$[M+H]^+$	-	-	11.29	LC-QTOF-MS	ESI +	3	0.69	4.22×10^{-2}	1.25
Tripeptide (Gly Thr Asp)	$C_{10}H_{17}N_3O_7$	291.1067	1.11	5	$[M-H-H_2O]^-$	-	-	15.41	LC-QTOF-MS	ESI -	3	0.59	3.17×10^{-3}	1.56
Tripeptide (Ala Asp Cys)	$C_{10}H_{17}N_3O_6S$	307.0838	1.11	5	$[M-H]^-$	-	-	5.68	LC-QTOF-MS	ESI -	3	0.60	1.17×10^{-3}*	1.82
Tripeptide (Cys Asp Ala)	$C_{10}H_{17}N_3O_6S$	307.0838	7.84	5	$[M-H]^-$	-	-	5.41	LC-QTOF-MS	ESI -	3	0.61	9.46×10^{-4}*	1.89
Organooxygen compounds														
dCDP	$C_9H_{15}N_3O_{10}P_2$	387.0233	9.62	0	$[M-H]^-$	-	-	8.63	LC-QTOF-MS	ESI -	3	0.65	5.27×10^{-3}	1.38
Pyrimidines and pyrimidine derivatives														
Carboxy-hydroxypropyl thiamine diphosphate	$C_{16}H_{25}N_4O_{10}P_2S$	527.0767	9.4	8	$[M+HCOOH-H]^-$	-	-	5.22	LC-QTOF-MS	ESI -	3	0.78	-	1.06
Pyridines and derivatives														
Nicotinamide	$C_6H_6N_2O$	122.0480	9.88	0	$[M-H]^-$	-	-	17.17	LC-QTOF-MS	ESI +	2	0.63	-	1.06

RT: retention time; [a]CV, coefficient of variation in the metabolites in the QC samples; [b]Metabolites were annotated with identification confidence levels as recommended by the Metabolomics Standards Initiative (MSI) according to: Level 0: Unambiguous 3D Structure: isolated, pure compound, including full stereochemistry; Level 1: Confident 2D structure: uses reference standard match or full 2D structure elucidation; Level 2: Probable structure: matched to literature data or databases by diagnostic evidence, Level 3: Possible structure or class: most likely structure, isomers possible, substance class or substructure match; Level 4: Unknown feature of interest [1]; [c]Fold Change, change in the abundance of the specified comparison calculated as (BLEGI-treated cells/untreated cells); [d]p value* corresponding to the *p* values calculated by the Benjamini-Hochberg false discovery rate post hoc correction (FDR < 0.05); [e]VIP, variable importance in projection. [f]Overlapped. LC: liquid chromatography, QTOF-MS: quadrupole time-of-flight mass spectrometer, ^{1}H-NMR: proton nuclear magnetic resonance, br s: broad singlet, integrates for one proton.

Table S2. List of all metabolites identified in the exometabolome of cancer cells HCT-116 treated with BLEGI.

Compounds	Formula	Mass	RT (min)	Error (ppm)	Adduct	δ ^{1}H (ppm) and Multiplicity, *J* in Hz	Assignments	[a]CV for QC (%)	Analytical platform	DET	[b]ID Level	BLEGI-treated cells vs. untreated cells		
												[c]Fold Change	[d]*p* value*	[e]VIP
Alcohols and polyols														
Myo-inositol	$C_6H_{12}O_6$	180.0634	1.14	10	$[M-H]^-$	-	-	10.59	LC-QTOF-MS	ESI -	3	1.21	-	1.29
Amino acid and derivatives														
Acetyl-glutamate	$C_7H_{11}NO_5$	189.0637	1.19	9	$[M-H]^-$	-	-	7.00	LC-QTOF-MS	ESI -	2	1.50	6.52×10^{-3}	1.58
Trimethyl -Lysine	$C_9H_{20}N_2O_2$	188.1525	3.86	9	$[M+Na]^+$	-	-	12.08	LC-QTOF-MS	ESI +	3	1.78	2.58×10^{-2}	-
Carboxylic acids and derivatives														
3-(3'-Methylthio)propylmalic acid	$C_8H_{14}O_5S$	222.0562	8.48	2	$[M+H]^+$	-	-	8.61	LC-QTOF-MS	ESI +	3	0.62	-	1.14
Phosphoglycerol-glutathione	$C_{13}H_{24}N_3O_{11}PS$	461.0869	1.16	3	$[M-H]^-$	-	-	15.65	LC-QTOF-MS	ESI -	3	0.72	-	1.27
Capryloyl glycine	$C_{10}H_{19}NO_3$	201.1365	5.06	9	$[M+Na]^+$	-	-	12.37	LC-QTOF-MS	ESI +	3	1.29	-	1.29
Carnitines														

Decanoyl-carnitine	$C_{17}H_{33}NO_4$	315.2410	6.28	2	$[M+H]^+$	-	-	12.59	LC-QTOF-MS	ESI +	2	1.41	3.06×10^{-2}	1.47
Fatty acids														
2,4-Decadienoic isobutyl amide	$C_{14}H_{25}NO$	223.1936	9.67	8	$[M+Na]^+$	-	-	15.04	LC-QTOF-MS	ESI +	2	0.74	3.03×10^{-2}	-
3-hydroxy-tetradecanedioic acid	$C_{14}H_{26}O_5$	274.1780	0.58	4	$[M+K]^+$	-	-	9.35	LC-QTOF-MS	ESI +	3	0.72	3.38×10^{-2}*	-
2-methylideneglutaric acid	$C_6H_8O_4$	144.0423	2.83	2	$[M+H-H_2O]^+$	-	-	2.94	LC-QTOF-MS	ESI +	2	0.62	8.21×10^{-3}	1.50
2-hydroxyglutaric acid	$C_5H_8O_5$	148.0372	9.68	2	$[M+H]^+$	-	-	19.93	LC-QTOF-MS	ESI +	3	0.57	-	1.20
Acetyl dihydrolipoamide	$C_{10}H_{19}NO_2S_2$	249.0857	0.79	8	$[M+HCOOH-H]^-$	-	-	11.85	LC-QTOF-MS	ESI -	3	1.29	-	1.15
Lauroleic acid	$C_{12}H_{22}O_2$	198.1620	6.84	9	$[M+HCOOH-H]^-$	0.90 (t, J = 6.96 Hz)<br>1.30 (br s)	t-CH_3<br>$(CH_2)n$	13.08	LC-QTOF-MS / ^1H-NMR	ESI - / ^1H	2	0.74	-	1.29
5-hydroxy capric acid	$C_{10}H_{20}O_3$	188.1412	6.63	8	$[M-H]^-$	-	-	9.87	LC-QTOF-MS	ESI -	2	1.92	4.73×10^{-4}*	2.30
HpODE	$C_{18}H_{32}O_4$	312.2301	8.78	7	$[M-H]^-$	-	-	18.70	LC-QTOF-MS	ESI -	3	1.43	2.17×10^{-2}	-
10-oxo-13-hydroxy-11-octadecenoic acid	$C_{18}H_{32}O_4$	312.2301	6.05	7	$[M-H]^-$	0.89 (t, J = 6.95 Hz)<br>1.35 (br s)	t-CH_3<br>$(CH_2)n$	12.59	LC-QTOF-MS / ^1H-NMR	ESI - / ^1H	3	1.78	2.68×10^{-2}	1.02
Glycerophospholipids														
GPC	$C_8H_{21}NO_6P$	258.1106	3.72	5	$[M-H-H_2O]^-$	-	-	5.72	LC-QTOF-MS	ESI +	3	1.36	3.31×10^{-2}	1.30
LPC 18:1	$C_{26}H_{52}NO_7P$	521.3481	9.17	3	$[M+Cl]^-$	-	-	11.91	LC-QTOF-MS	ESI -	2	1.29	-	1.16
LPE 20:0	$C_{25}H_{52}NO_7P$	509.3481	9.45	1	$[M+H]^+$	-	-	10.89	LC-QTOF-MS	ESI +	3	0.64	3.03×10^{-2}	1.37
PI 19:0	$C_{28}H_{55}O_{12}P$	614.3431	7.29	8	$[M+HCOOH-H]^-$	-	-	9.04	LC-QTOF-MS	ESI -	3	0.67	4.59×10^{-2}	-
PKODiA-PA	$C_{27}H_{47}O_{11}P$	578.2856	4.37	4	$[M+K]^+$	-	-	12.31	LC-QTOF-MS	ESI +	3	1.29	1.45×10^{-2}*	1.15
Organooxygen compounds														
Guanidino-3-amino-1,3-dideoxy-scyllo-inositol	$C_7H_{16}N_4O_4$	220.1172	0.62	6	$[M+H]^+$	-	-	19.42	LC-QTOF-MS	ESI +	3	1.52	1.76×10^{-2}	1.38
2-Hydroxybenzaldehyde	$C_7H_6O_2$	122.0368	9.37	2	$[M+H-H_2O]^+$	-	-	19.66	LC-QTOF-MS	ESI +	3	0.67	-	1.16
Peptides														
Dipeptide (Tyrosyl-Methionine)	$C_{14}H_{20}N_2O_4S$	312.1144	3.94	7	$[M+Na]^+$	-	-	8.92	LC-QTOF-MS	ESI +	3	1.52	3.00×10^{-2}*	-
Dipeptide (Leu-Met-OH)	$C_{16}H_{22}N_2O_6S$	370.1199	4.47	1	$[M+H-H_2O]^+$	-	-	7.68	LC-QTOF-MS	ESI +	3	1.26	3.91×10^{-2}	1.29
Dipeptide (Valyl-Glutamate)	$C_{10}H_{17}N_2O_5$	245.1137	0.62	8	$[M+H]^+$	-	-	9.31	LC-QTOF-MS	ESI +	3	1.26	-	1.24
Dipeptide (Arginyl-Serine)	$C_9H_{19}N_5O_4$	261.1437	0.81	8	$[M+K]^+$	-	-	12.07	LC-QTOF-MS	ESI +	3	1.37	-	1.14
Dipeptide (Glu-Pro)	$C_{10}H_{16}N_2O_5$	244.1059	2.36	5	$[M-H-H_2O]^-$	-	-	14.65	LC-QTOF-MS	ESI -	3	1.75	1.84×10^{-2}*	1.74
Dipeptide (Thr-Trp-OH)	$C_{21}H_{21}N_3O_7$	427.1379	4.41	5	$[M-H-H_2O]^-$	-	-	8.34	LC-QTOF-MS	ESI -	3	1.41	1.24×10^{-2}	1.73
Dipeptide (Nap-Thr-OH)	$C_{22}H_{20}N_2O_7$	424.1270	5.35	4	$[M-H]^-$	-	-	14.98	LC-QTOF-MS	ESI -	3	1.29	-	1.04
Tripeptide (Asn Cys Cys)	$C_{10}H_{18}N_4O_5S_2$	338.0719	0.74	7	$[M+Na]^+$	-	-	17.52	LC-QTOF-MS	ESI +	3	0.74	-	1.08

Tripeptide (Arg Trp Ala)	$C_{20}H_{29}N_7O_4$	431.2281	7.86	5	$[M+H]^+$	-	-	3.86	LC-QTOF-MS	ESI +	3	1.34	-	1.20
Tripeptide (His Lys Met)	$C_{17}H_{30}N_6O_4S$	414.2049	7.86	3	$[M+Na]^+$	-	-	5.01	LC-QTOF-MS	ESI +	3	1.32	-	1.04
Tripeptide (Phe-Trp-Gly)	$C_{22}H_{24}N_4O_4$	408.1798	4.91	1	$[M-H]^-$	-	-	12.42	LC-QTOF-MS	ESI -	3	0.64	1.52×10^{-2}	1.24
Tripeptide (Asn-Cys-Tyr)	$C_{16}H_{22}N_4O_6S$	398.1260	0.67	1	$[M+HCOOH-H]^-$	-	-	12.06	LC-QTOF-MS	ESI -	3	1.55	4.70×10^{-2}*	1.34
Tripeptide (Met-Pro-Pro)	$C_{15}H_{25}N_3O_4S$	343.1566	9.00	3	$[M+HCOOH-H]^-$	-	-	7.07	LC-QTOF-MS	ESI -	3	0.80	-	1.03
Nucleosides, nucleotides, and analogues														
Ribosylzeatin phosphate	$C_{15}H_{22}N_5O_8P$	431.1206	4.54	1	$[M+Na]^+$	-	-	13.69	LC-QTOF-MS	ESI +	3	0.71	9.49×10^{-3}*	1.50
Pyrimidines and pyrimidine derivatives														
Thymine	$C_5H_6N_2O_2$	126.0429	1.51	2	$[M+H]^+$	-	-	4.16	LC-QTOF-MS	ESI +	3	1.42	2.61×10^{-2}	1.41
Sugar and derivatives														
Cellobionate (4-Glucopyranosyl-gluconate)	$C_{12}H_{22}O_{12}$	358.1111	3.93	1	$[M+Na]^+$	-	-	2.40	LC-QTOF-MS	ESI +	2	1.21	1.20×10^{-2}*	1.53
Threonic acid	$C_4H_8O_5$	136.0372	1.07	9	$[M+H]^+$	-	-	1.74	LC-QTOF-MS	ESI +	3	0.75	-	1.30
Tryptamines														
Hydroxyl-tryptamine	$C_{10}H_{12}N_2O$	176.0950	2.83	3	$[M+H-H_2O]^+$	-	-	5.65	LC-QTOF-MS	ESI +	3	1.23	1.58×10^{-2}	1.56

RT: retention time; [a]CV, coefficient of variation in the metabolites in the QC samples; [b]Metabolites were annotated with identification confidence levels as recommended by the Metabolomics Standards Initiative (MSI) according to: Level 0: Unambiguous 3D Structure: isolated, pure compound, including full stereochemistry; Level 1: Confident 2D structure: uses reference standard match or full 2D structure elucidation; Level 2: Probable structure: matched to literature data or databases by diagnostic evidence, Level 3: Possible structure or class: most likely structure, isomers possible, substance class or substructure match; Level 4: Unknown feature of interest [1]; [c]Fold Change, change in the abundance of the specified comparison calculated as (BLEGI-treated cells/untreated cells); [d]p value* corresponding to the *p* values calculated by the Benjamini-Hochberg false discovery rate post hoc correction (FDR < 0.05); [e]VIP, variable importance in projection; [f]Overlapped. LC: liquid chromatography, QTOF-MS: quadrupole time-of-flight mass spectrometer, ^{1}H-NMR: proton nuclear magnetic resonance, br s: broad singlet, integrates for one proton.

Table S3. Retention times of chemical standards in the LC-MS analyses.

Chemical standard	Chemical class	UV λ_{max} (nm)	Molecular formula	t_R (min) LC-MS[a]	Exact mass	$[M-H]^-$	$[M+H]^+$
Gallic acid	Organic acid	275	$C_7H_6O_5$	1.09	170.0215	169.0132	-
Chlorogenic acid	Cinnamic acid and derivatives	275sh, 325	$C_{16}H_{18}O_9$	2.42	354.3095	353.0863	-
Caffeic acid	Cinnamic acid and derivatives	275	$C_9H_8O_4$	2.55	180.0422	179.0344	-
Isoorientin	Flavone glycosides	274, 355	$C_{21}H_{20}O_{11}$	2.96	448.3775	447.0954	449.1094
Ampelopsin	Flavanonol aglycones	300	$C_{15}H_{12}O_8$	2.88	320.0532	319.0458	-
Rutin	Flavonol glycosides	274, 355	$C_{27}H_{30}O_{16}$	3.20	610.1533	609.1531	611.1603
Vitexin	Flavone glycosides	273, 335	$C_{21}H_{20}O_{10}$	3.26	432.1056	431.0923	433.1123
p-Coumaric acid	Cinnamic acid and derivatives	295sh, 310	$C_9H_8O_3$	3.36	164.0473	163.0390	-
Sinapinic acid	Cinnamic acid and derivatives	325	$C_{11}H_{12}O_5$	3.78	224.2101	223.0684	-
Morin	Flavonol aglycones	264, 354	$C_{15}H_{10}O_7$	4.19	302.0426	301.0351	303.0512
Coumarin	Coumarins	275, 325	$C_9H_6O_2$	4.35	146.1432	-	147.0377
Quercetin	Flavonol aglycones	275, 358	$C_{15}H_{10}O_7$	4.49	302.0426	301.0344	303.0494
Cinnamic acid	Cinnamic acid and derivatives	275	$C_9H_8O_2$	4.63	148.1597	147.0524	-
Naringenin	Flavanone aglycones	275, 325	$C_{15}H_{12}O_5$	4.93	272.0684	271.0611	273.0764

[a] Column: ACQUITY UPLC HSS T3 C18 column (2.1 × 100 mm, 1.8 μm)

Table S4. Parameters used in the GNPS Classical Molecular Networking.

GNPS Classical Molecular Networking	
**Processing step**	**Parameters**
Basic options	Classical Molecular Networking<br>Precursor Ion Mass Tolerance: 0.02 Da<br>Fragment Ion Mass Tolerance: 0.02 Da
Advance Network Options	Min Pair Cos: 0.6<br>Network TopK: 10<br>Maximum Connected Component Size: 100<br>Minimun Matched Fragment Ions: 4<br>Minimum Cluster Size: 2<br>Maximum shift: 1999 Da<br>Run MSCluster: yes
Advance Library Search Options	Library Search Min Matched Peaks: 4<br>Search Analogs: Do Search<br>Score Threshold: 0.6<br>Maximum Analog Search Mass Difference: 100 Da

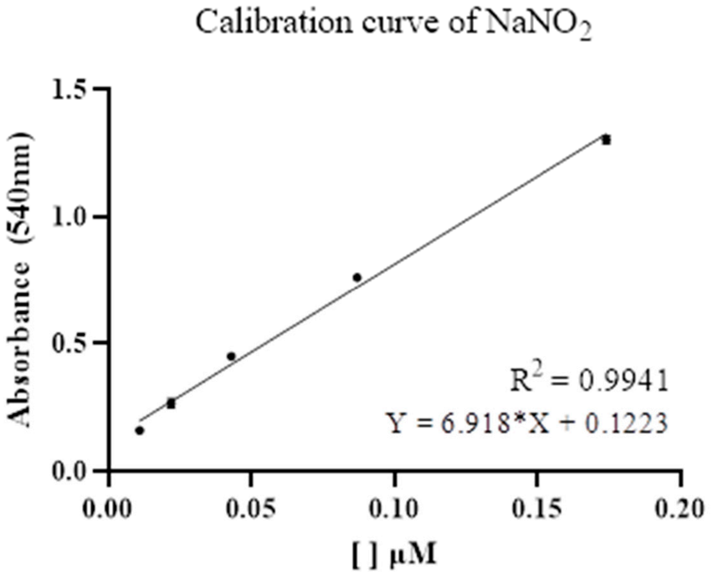

Figure S6. Calibration curve with sodium nitrite ($NaNO_2$) to represent the data in concentration of chemical NO_2^- formed.

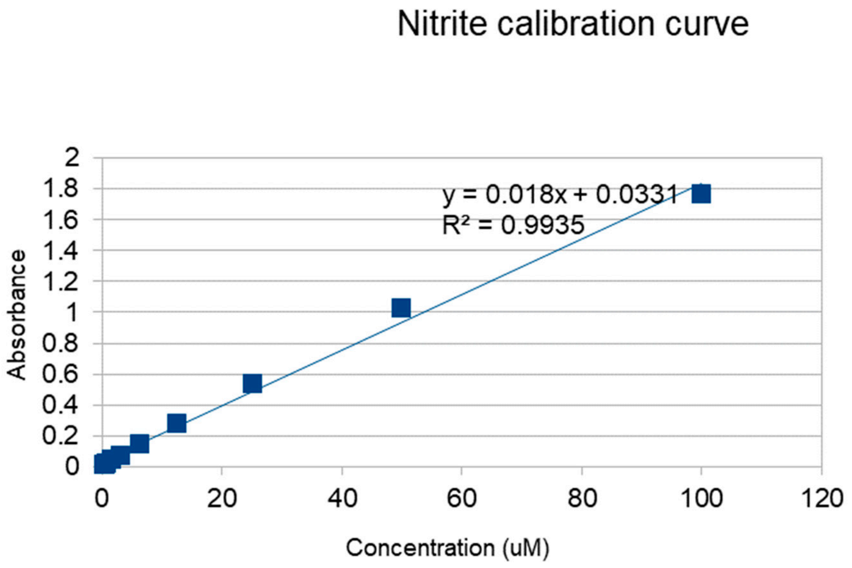

Figure S7. Calibration curve with sodium nitrite ($NaNO_2$) to represent the data in concentration of cellular NO_2^- formed.

References

1. Blaženović, I.; Kind, T.; Ji, J.; Fiehn, O. Software Tools and Approaches for Compound Identification of LC-MS/MS Data in Metabolomics. *Metabolites* 2018, *8*.
